# Revealing the immune cell subtype reconstitution profile in patients from the CLARITY study using deconvolution algorithms after cladribine tablets treatment

**DOI:** 10.1038/s41598-023-34384-5

**Published:** 2023-05-18

**Authors:** Irina Kalatskaya, Gavin Giovannoni, Thomas Leist, Joseph Cerra, Ursula Boschert, P. Alexander Rolfe

**Affiliations:** 1 EMD Serono Research & Development Institute, Inc. (an affiliate of Merck KGaA), 45 Middlesex Turnpike, Billerica, MA 01821 USA; 2 Blizard Institute, Barts and The London School of Medicine and Dentistry, Queen Mary University of London, London, UK; 3 Division of Clinical Neuroimmunology, Jefferson University, Comprehensive MS Center, Philadelphia, PA USA; 4 Ares Trading S.A. (an affiliate of Merck KGaA), Eysins, Switzerland; 5 Present Address: BISC Global, Boston, MA USA

**Keywords:** Computational biology and bioinformatics, Immunology

## Abstract

Immune Cell Deconvolution methods utilizing gene expression profiling to quantify immune cells in tissues and blood are an appealing alternative to flow cytometry. Our objective was to investigate the applicability of deconvolution approaches in clinical trial settings to better investigate the mode of action of drugs for autoimmune diseases. Popular deconvolution methods CIBERSORT and xCell were validated using gene expression from the publicly available GSE93777 dataset that has comprehensive matching flow cytometry. As shown in the *online tool*, ~ 50% of signatures show strong correlation (r > 0.5) with the remainder showing moderate correlation, or in a few cases, no correlation. Deconvolution methods were then applied to gene expression data from the phase III CLARITY study (NCT00213135) to evaluate the immune cell profile of relapsing multiple sclerosis patients treated with cladribine tablets. At 96 weeks after treatment, deconvolution scores showed the following changes vs placebo: naïve, mature, memory CD4^+^ and CD8^+^ T cells, non-class switched, and class switched memory B cells and plasmablasts were significantly reduced, naïve B cells and M2 macrophages were more abundant. Results confirm previously described changes in immune cell composition following cladribine tablets treatment and reveal immune homeostasis of pro- vs anti-inflammatory immune cell subtypes, potentially supporting long-term efficacy.

## Introduction

Peripheral whole blood is an accessible source of transcriptomic immune cell information for pharmacogenomic studies of human diseases, and is increasingly being incorporated into clinical studies.

Historically, methods such as flow cytometry^1^, and more recently, cytometry by time of flight (CyTOF)^2^ have been used for immune cell analysis. However, they have some limitations as they are costly, labour intensive, difficult to scale, and typically require fresh samples for analysis of some cell subtypes. In addition, a limited number of cell types can be detected, and it may not be possible for retrospective analysis of stored samples unless specifically prepared at the time of collection.

Immune cell deconvolution is an appealing alternative to flow cytometry for immune cell analysis that uses gene expression profiling such as microarray or RNA sequencing to quantify immune cells in blood and bulk tissue^3–7^. There are many well-described deconvolution methods that have been used for immune profiling in areas such as cancer and lupus^8,9^. Compared with flow cytometry, deconvolution methods provide additional details of underlying biological processes and gene expression data can be retrospectively analyzed. Flow cytometry datasets can be used to validate deconvolution outputs generated on gene expression data.

Multiple sclerosis (MS) is a complex disease with a dynamic variety of immune cells involved in its pathogenesis. Analyzing immune cells is important in understanding not only the pathogenesis of MS but also the mechanistic basis of drugs used to treat MS. Immune reconstitution therapies for MS result in transient immune reduction followed by immune repopulation, and the deconvolution method represents a tool for the investigation of these dynamics in the peripheral blood.

The phase III CLARITY study (CLAdRIbine Tablets treating multiple sclerosis orally; NCT00213135) was a 2-year, placebo-controlled study of cladribine tablets in relapsing MS^10^ that was successfully completed over 10 years ago. Cladribine tablets are an immune reconstitution therapy that acts via a selective, transient reduction of B and T cells followed by repopulation and corresponding immune reconstitution. Gene expression data from CLARITY are available for a subset of patients 96 weeks after the start of treatment (48 weeks after initiation of the second annual treatment cycle) for the two cladribine tablets doses in the study (cumulative dose of 3.5 mg/kg or 5.25 mg/kg over 2 years, henceforth referred to as cladribine tablets 3.5 mg/kg or cladribine tablets 5.25 mg/kg). Along with existing flow cytometry data these provide an opportunity to not only validate the deconvolution approach in the clinical setting but also to study the effect of cladribine tablets on reconstituting immune cell subsets^11,12^ not specifically studied in the original flow cytometry analysis (e.g. B cell and monocyte subtypes). Indeed, naïve B cell increases during the post-treatment phase could provide evidence of resetting of the immune system, contributing to immune homeostasis of pro- vs anti-inflammatory immune cell subtypes that potentially underscore the long-term efficacy of cladribine tablets.

The aim of the current analysis, therefore, was an initial validation of the popular deconvolution methods xCell^6^ and CIBERSORT^7^ with subsequent application to the CLARITY dataset. These two algorithms were selected as they are conceptually very different from each other. CIBERSORT is based on support vector regression model, and xCell is a signature based method that uses ssGSEA approach to score each sample (see methods). The validation was performed using flow cytometry and matched gene expression data from the publicly available rheumatoid arthritis GSE93777 dataset^13^. This dataset aids initial validation of the deconvolution method as it contains an unprecedented collection of publicly available flow cytometry data (26 cell types in total), includes patients using different treatment regimens, and gene expression from matching samples is assessed using the same GeneChip™ Human Genome U133 Plus 2.0 Arrays as the CLARITY data. This combination gives a unique opportunity to validate two immune cell deconvolution approaches. The validation was followed by application of the deconvolution methods to characterize the immune cell population dynamics during the immune reconstitution phase following treatment with cladribine tablets in the CLARITY study.

## Methods

### GSE93777 validation dataset

The GSE93777 dataset (https://www.ncbi.nlm.nih.gov/geo/query/acc.cgi?acc=GSE93777) includes microarray gene expression profiling of whole blood and sorted immune cells from rheumatoid arthritis patients, some of whom had received drug treatment (methotrexate, infliximab, and tocilizumab). The GSE93777 dataset also includes healthy volunteers (Table S1). Extensive flow cytometry data for 26 immune cell types was retrieved from the dataset for use in this analysis^14^.

### CLARITY data subset

CLARITY was a phase III, double-blind, placebo-controlled study, the findings of which have been previously published^10^. Briefly, 1326 patients with relapsing MS were randomized (1:1:1) to receive cladribine tablets 3.5 mg/kg (n = 433) or 5.25 mg/kg (n = 456) or placebo (n = 437) over two treatments of 8–10 days per 48-week treatment cycle. Gene expression data (GeneChip™ Human Genome U133 Plus 2.0 Array, Affymetrix, California, US) in stored whole blood samples at 96 weeks were available for 189 patients (cladribine tablets 3.5 mg/kg, n = 62; cladribine tablets 5.25 mg/kg, n = 70; placebo, n = 57), and prepared according to standard protocols^15^. Microarray .cel files are available from the Gene Expression Omnibus (GEO) under accession GSE185773^16^.

Flow cytometry was performed at various time points, as described previously^17^, and was available for nine cell types: natural killer cells (CD16^+^/CD56^+^), B cells (CD19^+^), pan T cells (CD3^+^), T helper cells (CD4^+^), cytotoxic T cells (CD8^+^), naïve T cells (CD4^+^/CD45RA^+^), memory T cells (CD4^+^/CD45RO^+^), naïve cytotoxic T cells (CD8^+^/CD45RA^+^), and memory cytotoxic T cells (CD8^+^/CD45RO^+^). Myeloid and B-cell subtypes were not included other than the mature B cell marker, CD19. The 96-week flow cytometry data were used for the deconvolution CLARITY study validation.

### Gene expression data: deconvolution methods (Fig. 1)

**Figure 1:**
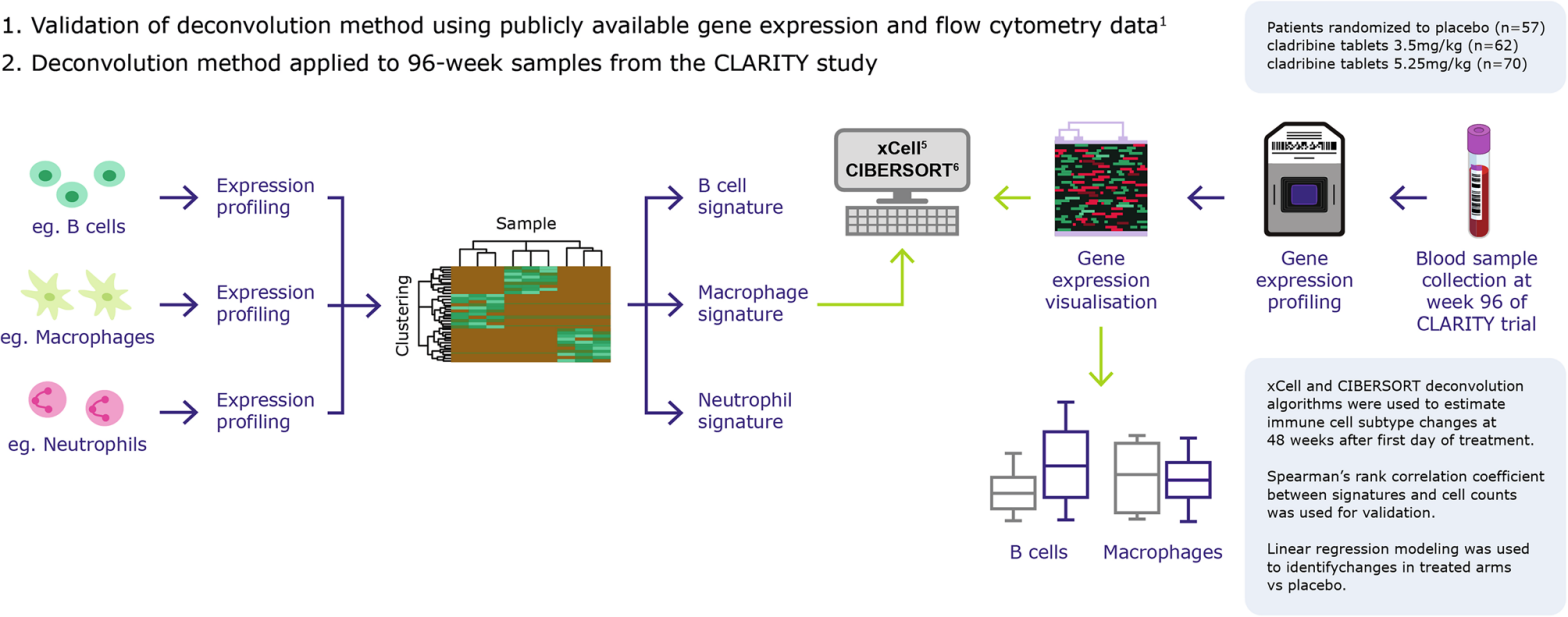
Schema of the overall study design. High level description of study design. The deconvolution method was validated using publicly available gene expression and flow cytometry data, then applied to 96 week samples from the CLARITY study.

Deconvolution methods comprise a system of equations that describe the expression of each gene in a heterogeneous sample as a combination of the expression levels of that gene across the different cell subsets present in the sample, weighted by their relative cell fractions. The purpose of deconvolution is to use the system of equations as a model to determine the most likely abundance or fraction of each cell type in a given sample. Different deconvolution methods are trained on different datasets (typically datasets of cells sorted by flow cytometry and then profiled using microarrays or RNA sequencing) and different algorithms are used to estimate the immune cell quantities.

### CIBERSORT

CIBERSORT deconvolution provides quantification for 22 immune cell subtypes. It is a widely used tool that requires an input matrix of reference gene expression signatures, collectively used to estimate the relative proportions of each cell type of interest^6^. To deconvolve the mixture, CIBERSORT uses a linear v-support vector regression model to find **f** in **m** = **f** x **B,** where **m** is a vector consisting of gene expression values from the mixture (e.g. bulk tumor or whole blood), **f**—a vector of fraction for each cell type, and **B** is a ‘signature matrix’ containing signature genes for each cell type^6^ A original leukocyte gene signature matrix, termed LM22, was used in this study as i contains 547 genes that distinguish 22 human immune cell subtypes. The CIBERSORT algorithm descripted in the Newman et al.^6^ paper was reimplemented using the svm method from the e1071 R package and original LM22 matrix. The term fraction is defined for each sample as the sum of values across the 22 cell subtypes with a total of 1 and should reflect the true fraction of cells in the sample of a given type. In this analysis, the CIBERSORT deconvolution method was performed on all 22 immune cell subtypes.

### xCell^7^

xCell deconvolution is a computational method that uses gene signatures to infer the abundance of 64 cell types including immune cell types. It is based on a single sample gene set enrichment analysis (ssGSEA) of ~ 10,000 genes and 489 gene signatures extracted from large-scale expression data from six projects (FANTOM, BluePrint, ENCODE, IRIS, HPCA, and Noverstern). This approach is based on ranking of each gene signature in the bulk tissue, then calculation of the enrichment score and spillover compensation to distinguish closely related cell types^7^.

For the present analysis, 20 unrelated signatures that represent cell types outside of the blood stream were identified and excluded as per the user manual (chondrocytes, osteoblasts, myocytes, keratinocytes, hepatocytes, endothelial cells, astrocytes, adipocytes, epithelial cells, mv and ly endothelial cells, neurons, pericytes, preadipocytes, skeletal muscle, sebocytes, mesangial cells, melanocytes, ly endothelial cells, smooth muscle, and fibroblasts). xCell was performed using the remaining set of 44 cell type signatures. Immune cell scoring was done using xCell R package v.1.1.

### GSE93777 dataset validation of deconvolution methods vs flow cytometry

The GSE93777 dataset^13^ was used for the initial validation of the deconvolution methods. Each sample was analyzed by xCell and CIBERSORT. Spearman’s rank correlation coefficient test was used to estimate the correlation (r) between deconvolution outputs and corresponding flow cytometry cell counts^18^. For this analysis we considered r > 0.5 = strong correlation, 0.3 < r < 0.5 = moderate correlation, r < 0.3 [false discovery rate (FDR) < 0.1] = weak correlation, and FDR > 0.1 = no correlation. Only relative estimates (% of total) for every cell type were used for the correlation studies. For all GSE93777 and CLARITY deconvolution signatures see Table S2.

### Online deconvolution and flow cytometry visualization tool (Rshiny app)

We designed an interactive web-based framework using Rshiny technology providing full access to all anonymized data and visualization material for the deconvolution cell signature and flow cytometry validation described in this article, available here: https://emdserono1.shinyapps.io/Immune_Cell_Deconvolution_Validation/). The framework includes an introduction to the publicly available dataset used for validation (GSE93777), a frequently asked questions section, and CIBERSORT and xCell outputs to allow the deconvolution findings of interest and corresponding flow cytometry data to be interactively selected and plotted. In addition, full access to numeric signature scoring is provided through interactive tables for user convenience as well as summary tables. In all cases, Spearman correlation is calculated, but the Pearson correlation coefficient approach can also be used. This tool can potentially be used by the broader scientific community for determining whether a specific immune cell signature could be reliably used for immune cell deconvolution in other patient subpopulation studies.

### CLARITY subset data validation of deconvolution methods vs flow cytometry

Spearman’s rank correlation coefficient test was used to estimate the correlation between deconvolution cell signatures and corresponding flow cytometry counts. Deconvolution cell signatures that did not map to corresponding or related immune cell subtypes assessed by flow cytometry were removed from this analysis. *P*-values were adjusted for multiple testing using the Bonferroni correction. All data visualization and statistical analyses were done in R.

### Deconvolution cell signature alterations after treatment with cladribine tablets in CLARITY data

Immune cell analysis of the CLARITY subset using the deconvolution cell signatures was undertaken for the cladribine tablets 3.5 mg/kg and 5.25 mg/kg treatment groups, separately and combined, and placebo. A multivariate linear regression model was built for each cell type to test whether the treatment arm is significantly related to the deconvolution score. Patients’ age and gender were used as covariates in the model. The *p* value from F-statistics was used to determine whether the relationship wa still significant when the model ccounted for covariates. Cell types for which both the adjusted *p*-values for the treatment coefficient and overall f-test *p*-value were < 0.1 were considered significant. lm() in R was used for linear regression modeling.

### Ethics approval and consent to participate

This retrospective study used data from the CLARITY study (NCT00213135), which was undertaken in compliance with the Declaration of Helsinki and standards of Good Clinical Practice according to the International Conference on Harmonisation of Technical Requirements for Registration of Pharmaceuticals for Human Use. At each centre, the relevant institutional review board or independent ethics committee reviewed and approved the trial protocol, patient information leaflet, informed consent forms, and investigator brochure (Table S6). All patients provided written informed consent to participate in the trials.

## Results

### GSE93777 dataset validation of deconvolution methods vs flow cytometry

All GSE93777 samples were used in the validation (rheumatoid arthritis patients with or without drug treatment and healthy volunteers). Treated rheumatoid arthritis patients received either methotrexate, infliximab, or tocilizumab in roughly equal parts. The majority of individuals in the GSE93777 dataset were female (226/255; 88.6%). The mean age was 54.9 years (Table S1).

CIBERSORT and xCell deconvolution cell signatures were mapped to the available flow cytometry data in order that deconvolution cell signatures could be compared to the flow cytometry results (Tables 1 and 2). Not every deconvolution cell signature was mapped precisely to the corresponding flow cytometry immune cell subtype (demonstrating the broad collection of signatures in the GSE93777 dataset); in addition, not all flow cytometry data were used for validation as some cell types (e.g. Bregs) are not yet provided by the deconvolution methods.

**Table 1:** Pairwise comparison between flow cytometry data from GSE93777 and corresponding CIBERSORT deconvolution cell signatures.

Cell signature	Flow cytometry cell phenotype	Spearman correlation (r)	*P*-value	FDR	Assessment
cibersort.B.cells.memory	B.CELL.rWBC	0.519	< 0.001	0.000	Strong correlation
cibersort.B.cells.naive	B.CELL.rWBC	0.164	0.011	0.017	Weak correlation
cibersort.Dendritic.cells.resting	DC.rWBC	0.188	0.003	0.006	Weak correlation
cibersort.Eosinophils	EOSINOPHIL.rWBC	0.146	0.024	0.031	Weak correlation
cibersort.Mast.cells.resting	BASOPHIL.rWBC	0.148	0.022	0.031	Weak correlation
cibersort.Monocytes	MONOCYTE.rWBC	0.604	< 0.001	0.000	Strong correlation
cibersort.Neutrophils	NEUTROPHIL.rWBC	0.798	< 0.001	0.000	Strong correlation
cibersort.NK.cells.resting	NK.rWBC	0.583	< 0.001	0.000	Strong correlation
cibersort.Plasma.cells	PLASMABLAST.rWBC	0.610	< 0.001	0.000	Strong correlation
cibersort.T.cells.CD4.memory.resting	MEMORY.CD4.rWBC	0.079	0.222	0.247	No correlation
cibersort.T.cells.CD4.naive	NAIVE.CD4.rWBC	0.691	< 0.001	0.000	Strong correlation
cibersort.T.cells.CD8	CD8.T.rWBC	0.776	< 0.001	0.000	Strong correlation
cibersort.T.cells.gamma.delta	GAMMA.DELTA.T.rWBC	− 0.078	0.229	0.247	No correlation
cibersort.T.cells.regulatory.Tregs	TREG.rWBC	− 0.021	0.744	0.744	No correlation

r > 0.5 = strong correlation, 0.3 < r < 0.5 = moderate correlation, r < 0.3 [false discovery rate (FDR) < 0.1] = weak correlation, and FDR > 0.1 = no correlation. Remaining cell types were not tested due to the lack of the corresponding flow cytometry data.

**Table 2:** Pairwise comparison between flow cytometry data from GSE93777 and corresponding xCell deconvolution cell signatures.

Cell signature	Flow cytometry cell phenotype	Spearman correlation (r)	*P*-value	FDR	Assessment
xcell.B.cells	B.CELL.rWBC	0.768	< 0.001	0.000	Strong correlation
xcell.Basophils	BASOPHIL.rWBC	0.016	0.802	0.802	No or negative correlation
xcell.CD4.memory.T.cells	MEMORY.CD4.rWBC	0.434	< 0.001	0.000	Moderate correlation
xcell.CD4.naive.T.cells	NAIVE.CD4.rWBC	0.786	< 0.001	0.000	Strong correlation
xcell.CD4.T.cells	CD4.T.rWBC	0.789	< 0.001	0.000	Strong correlation
xcell.CD4.Tcm	MEMORY.CD4.rWBC	0.466	< 0.001	0.000	Moderate correlation
xcell.CD4.Tem	MEMORY.CD4.rWBC	0.523	< 0.001	0.000	Strong correlation
xcell.CD8.naive.T.cells	NAIVE.CD8.rWBC	− 0.141	0.029	0.035	No or negative correlation
xcell.CD8.T.cells	CD8.T.rWBC	0.850	< 0.001	0.000	Strong correlation
xcell.CD8.Tcm	MEMORY.CD8.rWBC	0.488	< 0.001	0.000	Moderate correlation
xcell.CD8.Tem	CD45RA.MEMORY.CD8.rWBC	0.450	< 0.001	0.000	Moderate correlation
xcell.cDC	MDC.rWBC	0.289	< 0.001	0.000	Weak correlation
xcell.Class.switched.memory.B.cells	B.CELL.rWBC	0.738	< 0.001	0.000	Strong correlation
xcell.DC	DC.rWBC	0.062	0.340	0.370	No or negative correlation
xcell.Eosinophils	EOSINOPHIL.rWBC	0.479	< 0.001	0.000	Moderate correlation
xcell.Memory.B.cells	B.CELL.rWBC	0.731	< 0.001	0.000	Strong correlation
xcell.Monocytes	MONOCYTE.rWBC	0.259	< 0.001	0.000	Weak correlation
xcell.Naive.B.cells	B.CELL.rWBC	0.786	< 0.001	0.000	Strong correlation
xcell.Neutrophils	NEUTROPHIL.rWBC	0.797	< 0.001	0.000	Strong correlation
xcell.NK.cells	NK.rWBC	0.411	< 0.001	0.000	Moderate correlation
xcell.NKT	NKT.rWBC	− 0.252	< 0.001	0.000	No or negative correlation
xcell.pDC	PDC.rWBC	0.086	0.187	0.213	No or negative correlation
xcell.Plasma.cells	PLASMABLAST.rWBC	0.520	< 0.001	0.000	Strong correlation
xcell.Tgd.cells	GAMMA.DELTA.T.rWBC	0.329	< 0.001	0.000	Moderate correlation
xcell.Tregs	TREG.rWBC	0.048	0.461	0.480	No or negative correlation

r > 0.5 = strong correlation, 0.3 < r < 0.5 = moderate correlation, r < 0.3 [false discovery rate (FDR) < 0.1] = weak correlation, and FDR > 0.1 = no correlation. Remaining cell types were not tested due to the lack of the corresponding flow cytometry data.

### CIBERSORT

Fourteen of 22 CIBERSORT cell signatures were mapped to corresponding or related immune cell subtypes assessed by flow cytometry from the GSE93777 dataset. Deconvolution cell signatures for neutrophils, monocytes, natural killer cells, CD8^+^ T cells, and memory B cells showed the strongest correlation with flow cytometry cell counts (r > 0.5). Deconvolution cell signatures for memory B cells showed a higher correlation (r = 0.519) than for naïve B cells (r = 0.164), although the comparisons were to total flow cytometry B cell counts and therefore suboptimal. Naïve CD4^+^ T cell deconvolution cell signatures correlated with naïve CD4^+^ flow cytometry cell counts (r = 0.691), but memory CD4^+^ had no correlation with corresponding memory CD4 cell counts (r = 0.079). Resting dendritic deconvolution cell signatures had a poor (r = 0.188), albeit statistically significant (*p* = 0.003), correlation with the corresponding flow cytometry cell count. No correlation was found between gamma delta T cells or Treg deconvolution cell signatures and corresponding flow cytometry cell counts, possibly because CIBERSORT estimated abundance at 0. No suitable flow cytometry counterparts were found for M0/M1/M2 macrophages or for follicular helper T cell deconvolution signatures. Plasma cells are rarely found in blood and flow cytometry data did not include such cell counts. However, we did observe that the plasma deconvolution cell signature had a good correlation with plasmablast cell counts (r = 0.610). While not a precise match, the deconvolution cell signature for resting mast cells showed no correlation or significance compared with basophil counts.

There are four CIBERSORT ‘activated’ signatures in the collection, generated by activating (lipopolysaccharide for dendritic cells, IL2 or IL15 for natural killer, etc.) parent cell types and gene expression assessment. ‘Activated’ cell types were not validated as there was no match in the flow cytometry dataset.

In summary, 7 of 14 (50%) validated CIBERSORT deconvolution cell signatures showed strong Spearman correlation (r > 0.5) with corresponding flow cytometry data; 4 of 14 (28%) showed weak but significant correlation; and 3 of 14 (21%) showed no correlation (FDR > 0.1). Eight CIBERSORT signatures were not tested (Table 1 and online correlation tool [visualisation of analyses described in this article]: https://emdserono1.shinyapps.io/Immune_Cell_Deconvolution_Validation/ [Fig. S1]).

### xCell

Twenty-five of 44 xCell signatures were mapped to corresponding or related immune cell subtypes assessed by flow cytometry from the GSE93777 dataset.

B-cell, neutrophil, pan CD8^+^, plasmablasts, eosinophil, and natural killer deconvolution cell signatures showed significant, positive, and moderate/strong correlation (r > 0.4) with corresponding flow cytometry cell counts. All CD4^+^ subtypes for deconvolution cell signatures and cell count correlated well, with correlation ranging from r = 0.434 to r = 0.789. Monocyte deconvolution cell signatures correlation with corresponding flow cytometry cell counts was positive (r = 0.604) and statistically significant (*p* < 0.001). Deconvolution cell signatures showed poor correlation with flow cytometry cell counts for basophils (r = 0.148) and Tregs (r = − 0.021). Both the xCell deconvolution cell signature collection and the GSE93777 flow cytometry dataset contain a variety of dendritic cells, including DC, aDC, iDC, cDC, and pDC. The xCell deconvolution cell signature for cDC was mapped to mDC cell counts and showed a moderate but positive correlation (r = 0.289). Neither pan DC (r = 0.062) or pDC (r = 0.086) deconvolution cell signatures showed significant correlation with flow cytometry cell counts. Tgd deconvolution cell signatures showed a moderate correlation with flow cytometry cell counts (r = 0.329). Stem cell, progenitor, macrophage, platelet, and erythrocyte signatures (M0/M1/M2) were not validated as there was no match in the flow cytometry dataset.

In summary, 17 of 25 (68%) xCell validated deconvolution cell signatures showed moderate to strong Spearman correlation (r > 0.3) with corresponding flow cytometry data; 2 of 25 (8%) showed weak but significant correlation, and 6 of 25 (24%) showed no correlation (FDR > 0.1). Nineteen xCell deconvolution signatures were not tested (Table 2 and online correlation tool [visualisation of analyses described in this article]: https://emdserono1.shinyapps.io/Immune_Cell_Deconvolution_Validation/ [Fig. S2]).

### CLARITY data subset

The demographic and clinical characteristics of the 1326 patients in the CLARITY study have been previously described^10^. The subset of 189 CLARITY patients in this analysis was broadly similar (Table S3). Gene expression data (GeneChip™ Human Genome U133 Plus 2.0 Array) in whole blood samples at 96 weeks were available from patients randomized to placebo (n = 57), cladribine tablets 3.5 mg/kg (n = 62), and cladribine tablets 5.25 mg/kg (n = 70).

### CLARITY subset data validation of deconvolution methods vs flow cytometry

**Figure 2:**
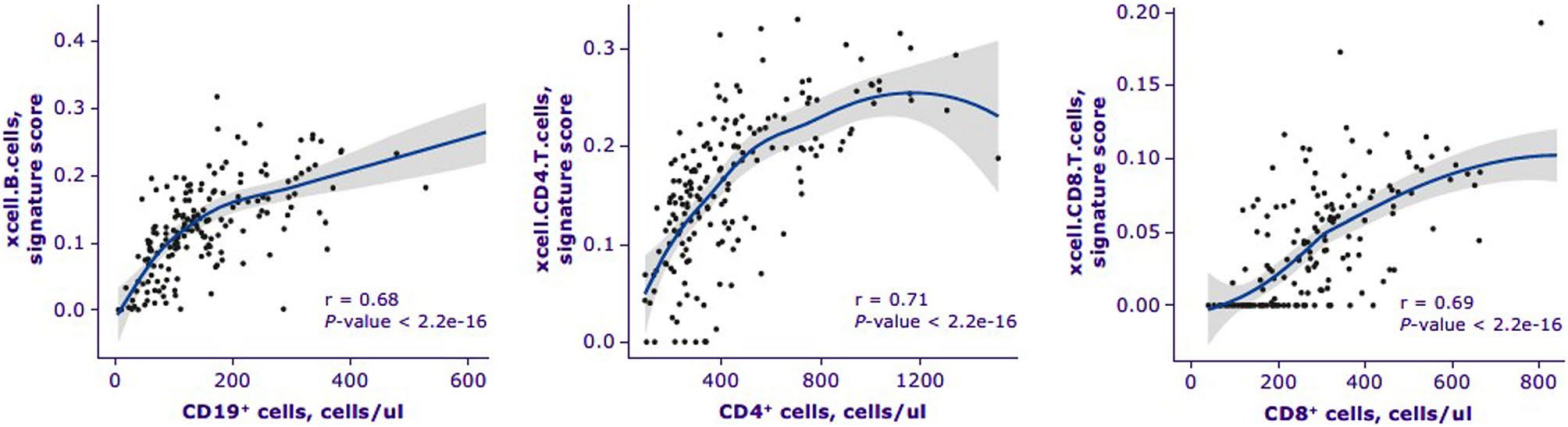
xCell deconvolution cell signature scores vs flow cytometry for major lymphocyte subtypes from CLARITY data. Spearman correlation is indicated.

Twenty-six out of 66 deconvolution cell signatures (9 CIBERSORT and 17 xCell) were matched to 1 of the 9 flow cytometry cell data at week 96 of the CLARITY study (Fig. 2, Table S4, Fig. S1). In contrast to the validation study, using the GSE93777 dataset, generic cell flow data were mapped to all related deconvolution signatures including activated forms (for example, there were no deconvolution cell signatures that represented pan T cells in human blood, so CD3^+^ flow cytometry cell counts were mapped to 17 deconvolution cell signatures including memory CD4^+^ T, Tregs, Tgd etc.).

**Figure 3:**
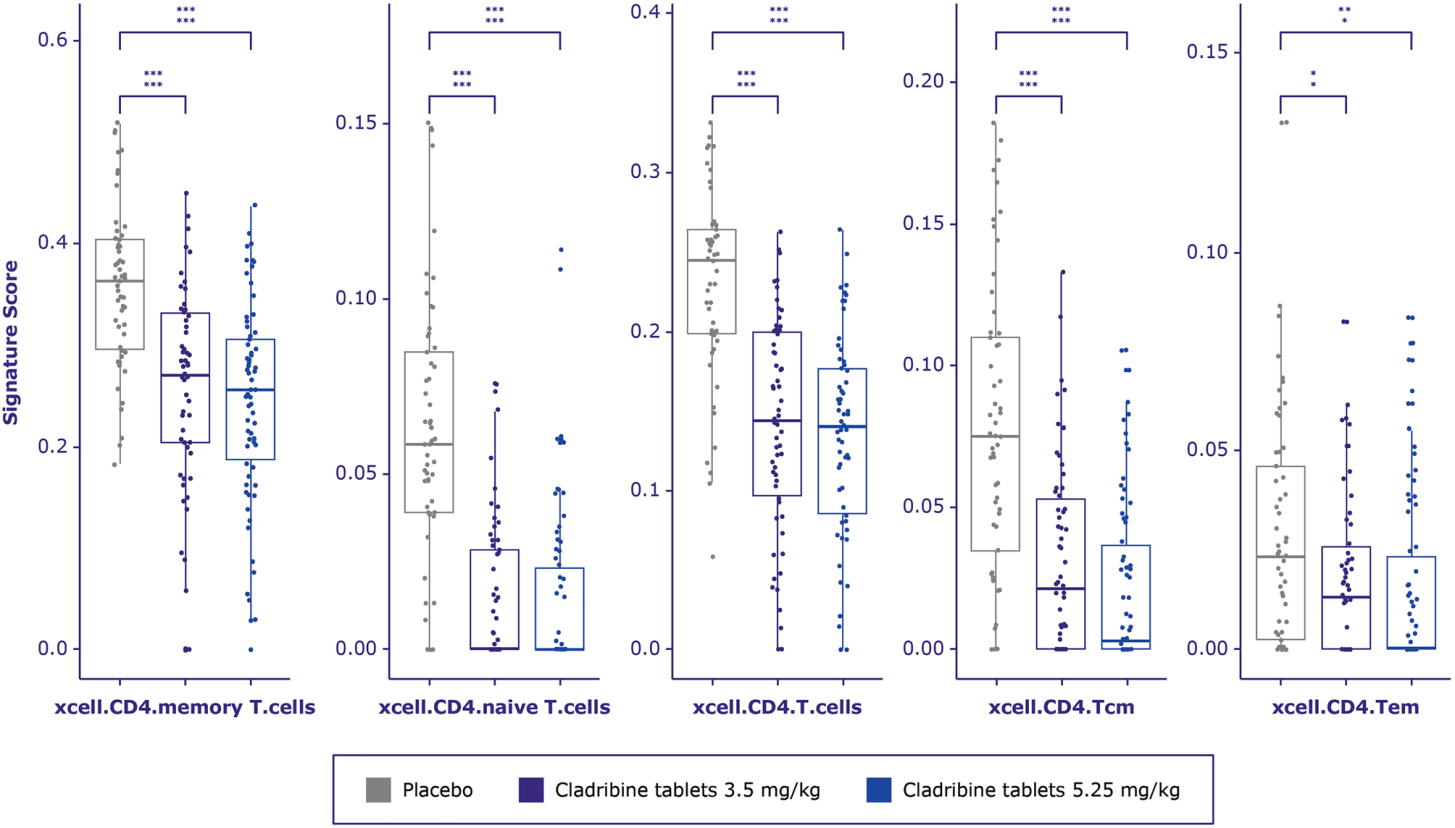
CD4^+^ T-cell signature score distribution generated by xCell between treatment arms in CLARITY. A multivariate linear regression model was built for each cell type to test whether treatment arm is significantly related to the deconvolution score. *P*-value from F-statistics was used to determine whether the relationship is still significant when model is accounted for patient’s age and gender. Cell types for which either the adjusted *p*-values for the treatment coefficient or overall F-test adjusted *p*-value were > 0.1 were considered not significant and marked as ns. Adjusted *p*-value from F-test (lower row) and adjusted *p*-value from linear model where only treatment arm was used as a covariate (upper row) are marked as asterisks * < 0.1, ** < 0.01, *** < 0.001. *cm* central memory; *em* effector memory

Fourteen of 52 comparisons showed adjusted *p*-values > 0.1. Three of the 14 comparisons represented an ‘activated’ form of the deconvolution cell signature, counts that are rarely detected by basic flow cytometry. The CD3^+^ flow cytometry cell counts had some correlation with the majority of deconvolution T cell signatures; however, cibersort.T.cells.regulatory.Tregs, xCell.Th1.cells, xCell.CD8 + .naive T-cells, cibersort.T.cells.CD4.memory.resting, and cibersort.T.cells.CD4.memory.activated are very specialized T cell subtypes. xCell deconvolution cell signature scores were strongly correlated with corresponding flow cytometry cell counts for CD19^+^, CD4^+^, and CD8^+^ T cells (Fig. 3).

The final dataset consisted of 63 deconvolution cell signatures generated for all 189 patients in this subset of the CLARITY study cohort: 19 from CIBERSORT and 44 from xCell. Over 99% of values in Mast.cells.activated, T.cells.follicular.helper and T.cells.gamma.delta signatures from CIBERSORT were estimated at 0, and therefore these signatures were excluded.

### Deconvolution cell signature alterations after treatment with cladribine tablets in CLARITY

Three comparisons were undertaken vs placebo: cladribine tablets 3.5 mg/kg dose, cladribine tablets 5.25 mg/kg dose, and combined dose. Patient’s age and gender are clinical factors that might affect immune cell abundance along with the treatment. That’s why Multivariate Linear Regression model was generated for every cell type using treatment arm, gender and age as independent variables. Cell types for which adjusted *p*-values from the treatment coefficient and overall adjusted significance from F-statistics <  = 0.1 were considered significant. This set up helped us to prioritize a list of the cell types affected by cladribine treatment but not patient’s gender or patient’s age attributes. Nine of 19 CIBERSORT and 24 of 44 xCell deconvolution cell signatures were altered in cladribine tablets treated patients vs placebo. There were significant alterations in 25 cell types for 3.5 mg/kg cladribine tablets vs placebo, and significant alterations in 31 cell types for 5.25 mg/kg cladribine tablets vs placebo (Table S5).

### T cells

**Figure 4:**
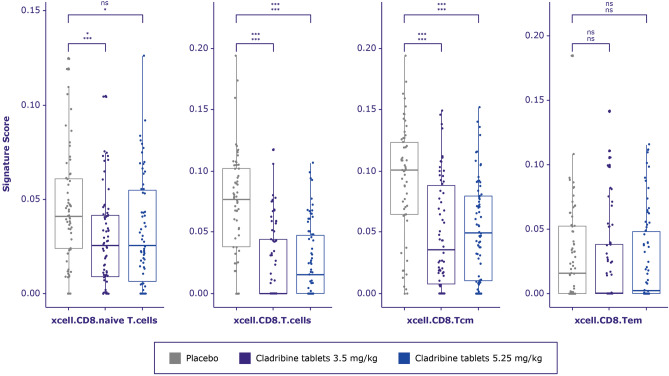
CD8 + T-cell signature score distribution generated by xCell between treatment arms in CLARITY. A multivariate linear regression model was built for each cell type to test whether treatment arm is significantly related to the deconvolution score. *P*-value from F-statistics was used to determine whether the relationship is still significant when model is accounted for patient’s age and gender. Cell types for which either the adjusted *p*-values for the treatment coefficient or overall F-test adjusted *p*-value were > 0.1 were considered not significant and marked as ns. Adjusted *p*-value from F-test (lower row) and adjusted *p*-value from linear model where only treatment arm was used as a covariate (upper row) are marked as asterisks * < 0.1, ** < 0.01, *** < 0.001.

Deconvolution cell signature scores for CD4^+^, CD4^+^ central memory, and CD4^+^ effector memory T cells were significantly decreased for both doses of cladribine tablets vs placebo at 96 weeks of the CLARITY study (Fig. 3). Deconvolution cell signature scores for CD8^+^ and CD8^+^ central memory cells were also significantly decreased for both doses of cladribine tablets vs placebo at 96 weeks. The changes in CD8^+^ effector cells were not significant (Fig. 4).

### B cells

**Figure 5:**
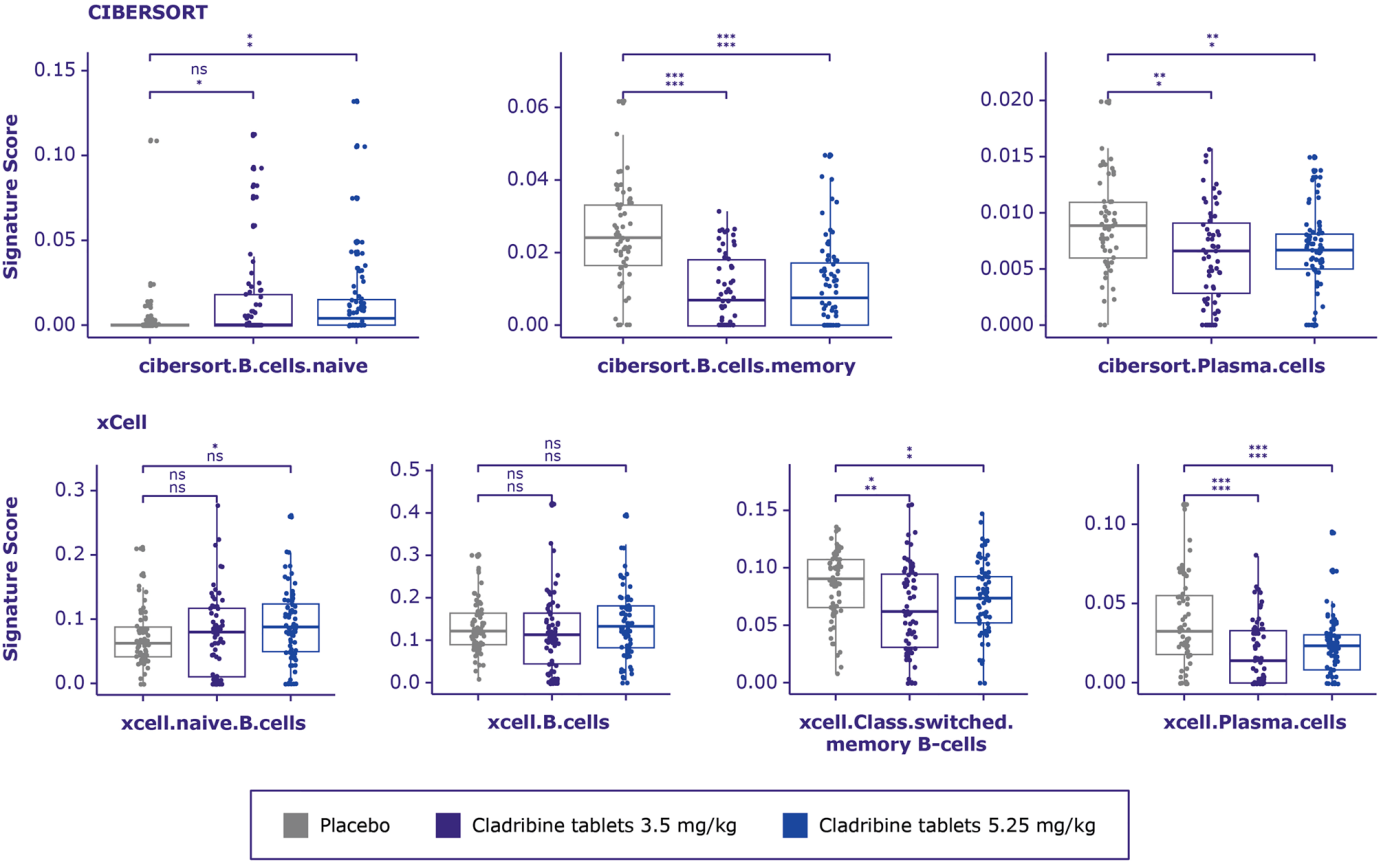
B cell signature score distribution generated by CIBERSORT and xCell between treatment arms in CLARITY**.** A multivariate linear regression model was built for each cell type to test whether treatment arm is significantly related to the deconvolution score. *P*-value from F-statistics was used to determine whether the relationship is significant when patient’s age and gender are used as covariates. Cell types for which either the adjusted *p*-values for the treatment coefficient or overall F-test adjusted *p*-value were > 0.1 were considered not significant and marked as ns. Adjusted *p*-value from F-test (lower row) and adjusted *p*-value from linear model where only treatment arm was used as a covariate (upper row) are marked as asterisks * < 0.1, ** < 0.01, *** < 0.001.

Deconvolution cell signature scores for class-switched memory, memory and plasmablasts were significantly decreased for both doses of cladribine tablets vs placebo at 96 weeks of the CLARITY study. Deconvolution cell signature scores for naïve B cells were significantly increased for 5.25 mg/kg cladribine tablets vs placebo using CIBERSORT deconvolution cell signatures (Fig. 5) and this trend stays significant if all treated samples are compared vs placebo (Table S5). It is interesting to note that naïve B cell signature derived using xCell method was also upregulated after high dose cladribine treatment (Fig. 5, lower panel), but only in the simple linear model with treatment arm as a covariate. If gender and age were taken into account, this significance is faded.

### Macrophages

**Figure 6:**
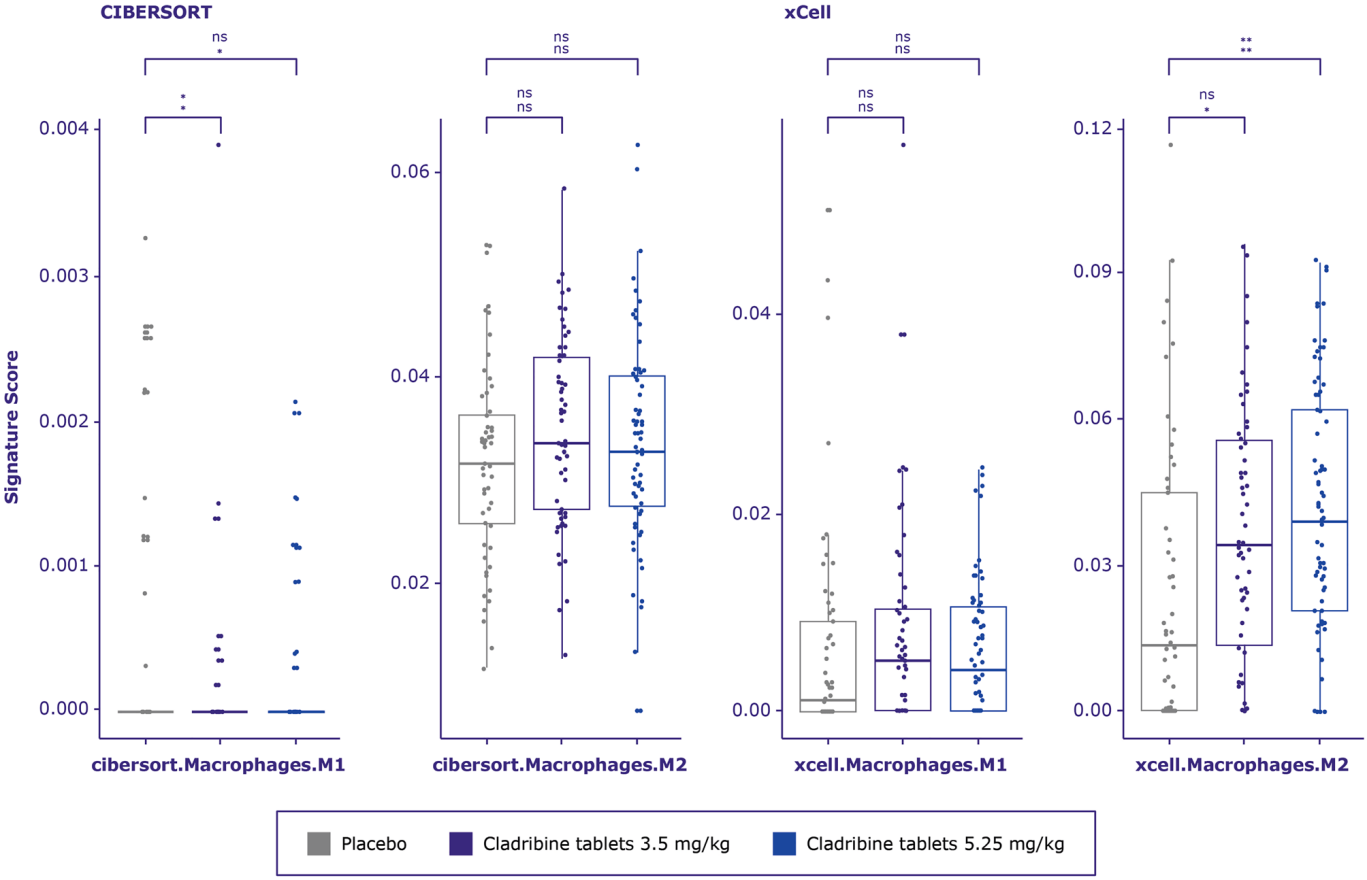
Monocyte and macrophage signature score distribution generated by CIBERSORT and xCell between treatment arms in CLARITY. A multivariate linear regression model was built for each cell type to test whether treatment arm is significantly related to the deconvolution score. *P*-value from F-statistics was used to determine whether the relationship is still significant when model is accounted for patient’s age and gender. Cell types for which either the adjusted *p*-values for the treatment coefficient or overall F-test adjusted *p*-value were > 0.1 were considered not significant and marked as ns. Adjusted *p*-value from F-test (lower row) and adjusted *p*-value from linear model where only treatment arm was used as a covariate (upper row) are marked as asterisks * < 0.1, ** < 0.01, *** < 0.001.

M2 macrophage scores from xCell and monocyte from CIBERSORT deconvolution cell signature significantly increased for those treated with high cladribine tablets (or all treated samples merged) vs placebo (Fig. 6, Table S5), whereas M1 macrophage signature from CIBERSORT was significantly depleted after low dose cladribine.

## Discussion

The speed of development of computational biology and bioinformatics provides additional opportunities to use mathematical algorithms to study complex human systems such as blood, with its heterogeneous mixture of multiple cell types. Immune cell deconvolution has been successfully used in multiple fields including cancer^9,14,19–21^ and immunity; for example, to analyse immune cells of the knee in osteoarthritis^22^ and the blood cell composition of patients with systemic lupus erythematosus^23^. Immune cell deconvolution methods, however, have not thus far been used to study blood samples from patients with MS.

In this analysis, the deconvolution methods xCell^6^ and CIBERSORT^7^ were successfully validated using existing flow cytometry and matched gene expression data from both the publicly available rheumatoid arthritis GSE93777 dataset^13^ and the CLARITY study^10^ dataset. Deconvolution methods were then applied to characterize the immune cell population dynamics from a subset of 189 patients from the CLARITY study with gene expression data at 96 weeks after the start of treatment. In line with previously described changes in immune cell composition, T cells and B cells were less abundant with cladribine tablets than with placebo. New results included that naïve B cells and macrophage M2 cells were more abundant whilst macrophage M1 cells showed signs of depletion compared with placebo, revealing immune homeostasis of pro- vs anti-inflammatory immune cell subtypes and potentially supporting long-term efficacy of cladribine tablets.

There are a variety of methods available for deconvolution, however, CIBERSORT and xCell methods were chosen for this analysis as they have previously performed with high effectiveness^6,7^. The aim of this validation was not to benchmark these methods versus other deconvolution methods, but because they were considered potentially effective for immunophenotyping MS disease. To validate such methods for use in this analysis, a publicly available dataset (rheumatoid arthritis GSE93777)^13^ was used to compare levels of immune cell signatures with corresponding flow cytometry. Good concordance of cell signatures between the CIBERSORT and xCell deconvolution methods were observed, similar to previous analyses describing immune cell levels in healthy liver and hepatocellular carcinoma^24^. Both CIBERSORT and xCell deconvolution methods showed significant, positive, and strong correlation for B cell, neutrophil, pan CD8^+^, plasmablasts, and natural killer cell deconvolution signatures (but poor correlation for basophils and Tregs) with corresponding flow cytometry cell counts. One reason the basophil deconvolution signatures were not as well correlated with flow cytometry may be because six of the nine basophil samples used for deconvolution cell signature generation^6^ were isolated from umbilical cord blood (Novershterm dataset: GSM609632, GSM609633, GSM609634, GSM609635, GSM609636, GSM609637), and may represent younger or otherwise different cells than those assessed in the flow cytometry data. While CIBERSORT and xCell deconvolution findings aligned for most cell types there were some differences that can partially be explained by the perspective of the mathematical method. Initially, both methods select the most representative sets of genes for each tissue type called gene signatures. Then, CIBERSORT deconvolution uses a linear v-support vector regression model^6^ and xCell uses s sSEA analysis^7^. Other differences include that CIBERSORT provides 22 immune cell subtypes and xCell provides 44 immune cell subtypes detected in whole blood. Other reasons for lack of correlation could have been because of an insufficient match in the flow cytometry collection. For example, for the CIBERSORT method, the match for resting mast cells (cibersort.Mast.cells.resting) was basophils. As well, some T cell-related signatures (cibersort.t.cells.regulatory and cibersort.t.cells.gamma.delta) produced zero values because of low sensitivity to rare cell subtypes and/or low counts for these cells in the peripheral blood (although it should be noted that this is not always the case, plasma cells were well correlated despite less than 1% in whole blood). General reasons for lack of correlation with either method, may also be due to the origin of the cells that were used for signature generation (in vivo vs in vitro; blood vs organ origin isolation). For example, dendritic cells used for CIBERSORT signature construction were generated by in vitro monocyte differentiation with IL4 + GMCSF stimulation, but not through direct isolation from the whole blood. As with basophils, eosinophil samples used for deconvolution cell signature generation were isolated from umbilical cord blood. None of the stem cell, progenitor, macrophage, platelet, or erythrocyte signatures (M0/M1/M2) were validated with either deconvolution method, as no matches were found in the GSE93777 flow cytometry dataset.

The deconvolution methods were used to evaluate immune cell types from stored blood samples of 189 patients with MS, a subset of the cohort of patients enrolled in the phase III efficacy study of cladribine tablets in MS (CLARITY). Strengths of using the GSE93777 and CLARITY datasets in the same analysis include that the majority of rheumatoid arthritis patients within the GSE93777 dataset were treated with highly efficacious drugs (methotrexate, infliximab, and tocilizumab) that may have shifted/altered their gene expression profiles. This highlights that deconvolution methods yield accurate results even after treatment with immunomodulatory drugs, increasing confidence in the results from the CLARITY subset analysis of patients having received two annual treatment cycles of cladribine tablets. In addition, the initial validation using the GSE93777 dataset was on the same sample type (whole blood) and microarray type (GeneChip™ Human Genome U133 Plus 2.0 Arrays) as the CLARITY data. Limitations of these analyses include that signatures generated on RNA sequencing rather than microarray gene expression analysis may reveal different results and would need to be validated and matched with flow cytometry in order to apply CIBERSORT or xCell deconvolution methods with full confidence. There were also some minor discrepancies, including lack of expected correction between the cibersort.T.Cells.CD4.memory.resting cell signature and CD4^+^/CD45RA flow cytometry cell count (similar results were observed with the GSE93777 validation). In addition, of the validated cell types, 21% (3 out of 14) of xCell and 24% (6 out of 25) of CIBERSORT deconvolution signatures showed no correlation with flow cytometry. In the future, these signatures will be used with caution or excluded from consideration.

Cladribine tablets in MS lead to immune reconstitution by a selective reduction in B and T cell counts, followed by a period of reconstitution, with clinical efficacy sustained beyond total lymphocyte recovery^17,25,26^. Available flow cytometry data has given valuable insight into B and T cells levels^11,12^, and early findings from ongoing studies in MS patients with high disease activity have shown a specific pattern of peripheral blood mononuclear cell subtype dynamics that coincide with the pattern of clinical efficacy in terms of early onset of action and sustained effect^25,27^. Thus far, immune cell levels at the two-year time point following cladribine tablets treatment has not been investigated in detail. The present study, therefore, represented an opportunity to not only validate the deconvolution approach but build upon the understanding of immune cell dynamics following completion of two cycles of cladribine tablets and after initial immune reconstitution, as well as compare differences between the 3.5 and 5.25 mg/kg doses analyzed in the CLARITY study. Using the validated deconvolution methods, consistent levels of cells were observed between doses (the 5.25 mg/kg dose did not show lower levels of immune cells than the 3.5 mg/kg dose) in line with previous observations from the CLARITY and CLARITY Extension studies and the clinical observation that the higher dose is not more effective^10,26^. In addition to immune cell levels previously evaluated by flow cytometry for those treated with cladribine tablets^28,29^, class switched and non-class switched memory B cells, naïve B cells, plasmablasts, and M1/M2 macrophages were analyzed in the present study. As with the GSE93777 dataset, deconvolution cell signatures from the CLARITY study were well correlated with flow cytometry data and are consistent with previous flow cytometry findings^17^. This supports the interpretation via deconvolution of levels of cell types not previously analyzed by flow cytometry and highlights the future research utility of this method for MS and other data. In this analysis, memory B cells, plasmablasts, total and memory CD4^+^ and CD8^+^ T cells and M1 macrophages were significantly reduced with cladribine tablets vs placebo, while naïve B cells and M2 macrophages were significantly increased. This positive change in M2 macrophages has not been previously reported, and may reflect a switch from M1 to M2 through the nuclear factor kappa B (NF-κB) or Nrf2 pathways^30,31^; in vitro studies also indicate that cladribine decreases the secretion of IL-6 and TNF-α^32^. Most of the reduced signatures were from T-/B-cell types and enhanced myeloid types. Thus, neutrophil signatures assessed by both methods showed upregulation, indicating that neutrophils to some extent replace the reduced lymphocytes. Stem and progenitor cells are not altered by cladribine tablets other than common lymphoid progenitor (CLP) cells. In addition, naïve B cells increased during the post-treatment phase, which could suggest a resetting of the immune system. Such findings warrant further validation in the clinical trial setting, including evaluation of the effect on immune cells for example 3 or 4 years after cladribine tablets initiation. A detailed analysis of immune cell subsets in patients with high disease activity could also yield useful comparisons.

The use of some disease-modifying therapies (DMTs) for MS may alter vaccine efficacy^33,34^, such as a reduced humoral response to inactivated vaccines^35,36^. However, cladribine tablets treatment has not been found to impair humoral response to COVID-19 vaccination^37,38^. The reconstitution of naïve B cell counts, as validated in the current analysis, may therefore serve to explain this finding.

## Conclusion

In summary, this study validates immune cell deconvolution as a reliable method for immune cell subtype assessment and shows new applicability in the analysis of immune cell data within the setting of relapsing MS, using data from the CLARITY study. The results confirm previously described changes in immune cell composition following treatment with cladribine tablets, and reveal immune homeostasis of pro- vs anti-inflammatory immune cell subtypes that potentially support long-term efficacy. In addition, first time gene expression profiling of cladribine-treated MS patients, along with placebo control, is made available for the broad scientific community through a GEO portal.

## Supplementary Information

Supplementary Information 1.

Supplementary Information 2.

Supplementary Information 3.

## Data Availability

Any requests for data by qualified scientific and medical researchers for legitimate research purposes will be subject to Merck’s Data Sharing Policy. All requests should be submitted in writing to Merck’s data-sharing portal https://www.merckgroup.com/en/research/our-approach-to-research-and-development/healthcare/clinical-trials/commitment-responsible-data-sharing.html. When Merck has a co-research, co-development, or co-marketing or co-promotion agreement, or when the product has been out-licensed, the responsibility for disclosure might be dependent on the agreement between parties. Under these circumstances, Merck will endeavour to gain agreement to share data in response to requests. The datasets generated and/or analysed during the current study are available in the Gene Expression Omnibus (GEO) repository, accession number: GSE185773 [https://www.ncbi.nlm.nih.gov/geo/query/acc.cgi?acc=GSE185773].

## Abbreviations

DC: Dendritic cells
CLARITY: CLAdRIbine tablets treating multiple sclerosis
CyTOF: Cytometry by time of flight
GEO: Gene expression omnibus
MS: Multiple sclerosis
ssGSEA: Single sample gene set enrichment analysis
SVR: Support vector regression
