# Supplementary material for: Revealing the immune cell subtype reconstitution profile in patients from the CLARITY study using deconvolution algorithms after cladribine tablets treatment
Source: Sci Rep. 2023 May 18;13:8067. doi: 10.1038/s41598-023-34384-5 (PMC10193326; doi:10.1038/s41598-023-34384-5)

**Supplementary Material**

**Table S1.** Baseline characteristics of the publicly available rheumatoid arthritis GSE93777 dataset that was used for immune cell deconvolution method evaluation.

|  | **Healthy volunteers (n=30)** | **Rheumatoid arthritis patients (n=209)** |
| --- | --- | --- |
| Sex, n (%)  Female  Male | 30 (100)  - | 186 (89.1)  23 (10.9) |
| Age, years  Mean (SD)  Median (range) | 39.3 (9.44)  41 (24–55) | 57.6 (14.9)  60 (25–82) |
| Treatment  Infliximab  Methotrexate  Tocilizumab  Treatment naïve | NA | 68 (28.5)  74 (31.0)  59 (24.7)  8 (3.3) |

NA, not applicable; SD, standard deviation.

**Table S3.** Baseline characteristics of the CLARITY (NCT00213135) subset of patients that was used for gene expression assessment.

|  | **Cladribine tablets 3.5 mg/kg (n=63)** | **Cladribine tablets 5.25 mg/kg (n=70)** | **Placebo (n=57)** | **Total (n=189)** |
| --- | --- | --- | --- | --- |
| Sex, n (%)  Female  Male | 44 (71.0)  18 (29.0) | 50 (71.4)  20 (28.6) | 32 (56.1)  25 (43.9) | 126 (66.7)  63 (33.3) |
| Age  Mean (SD)  Median (range) | 39.1 (10.1)  40 (19–62) | 40.5 (10.6)  42 (20–64) | 38.4 (9.7)  39 (19–58) | 39.4 (10.2)  40 (19–64) |

SD, standard deviation.

**Table S4.** Pairwise comparison between flow cytometry data from CLARITY (9 cell types) and corresponding or related CIBERSORT and xCell deconvolution cell signatures using Spearman correlation.

| **Phenotype** | **Signature** | **r** | **p-value** | **Bonferroni** |
| --- | --- | --- | --- | --- |
| CD16/CD56 | xcell.NK.cells | 0.3487 | 1.72E-06 | 8.92E-05 |
| CD16/CD56 | cibersort.NK.cells.resting | 0.4616 | 7.81E-11 | 4.06E-09 |
| CD16/CD56 | cibersort.NK.cells.activated | 0.2059 | 0.005674455 | 2.95E-01 |
| CD19 | xcell.Class-switched.memory.B.cells | 0.6443 | 2.24E-22 | 1.16E-20 |
| CD19 | xcell.Memory.B.cells | 0.5896 | 3.84E-18 | 2.00E-16 |
| CD19 | xcell.naive.B.cells | 0.6466 | 1.42E-22 | 7.39E-21 |
| CD19 | xcell.pro.B.cells | 0.0796 | 0.289022865 | 1.00E+00 |
| CD19 | cibersort.B.cells.naive | 0.4019 | 2.44E-08 | 1.27E-06 |
| CD19 | cibersort.B.cells.memory | 0.0374 | 0.618673719 | 1.00E+00 |
| CD3 | xcell.CD4.memory.T.cells | 0.6078 | 1.84E-19 | 9.58E-18 |
| CD3 | xcell.CD4.naive.T.cells | 0.5144 | 1.76E-13 | 9.16E-12 |
| CD3 | xcell.CD4.T.cells | 0.7214 | 4.70E-30 | 2.44E-28 |
| CD3 | xcell.CD4.Tcm | 0.4756 | 1.71E-11 | 8.90E-10 |
| CD3 | xcell.CD4.Tem | 0.2377 | 0.001354215 | 7.04E-02 |
| CD3 | xcell.CD8.naive T.cells | 0.1469 | 0.049619043 | 1.00E+00 |
| CD3 | xcell.CD8.T.cells | 0.6164 | 4.04E-20 | 2.10E-18 |
| CD3 | xcell.CD8.Tcm | 0.5649 | 1.76E-16 | 9.14E-15 |
| CD3 | xcell.CD8.Tem | 0.2804 | 0.000143038 | 7.44E-03 |
| CD3 | xcell.Th1.cells | -0.0283 | 0.706790539 | 1.00E+00 |
| CD3 | xcell.Th2.cells | 0.2569 | 0.000516056 | 2.68E-02 |
| CD3 | xCell.Tregs | 0.3003 | 4.41E-05 | 2.29E-03 |
| CD3 | cibersort.T.cells.CD8 | 0.3825 | 1.26E-07 | 6.53E-06 |
| CD3 | cibersort.T.cells.CD4.naive | 0.3181 | 1.43E-05 | 7.41E-04 |
| CD3 | cibersort.T.cells.CD4.memory.resting | 0.0143 | 0.848860479 | 1.00E+00 |
| CD3 | cibersort.T.cells.CD4.memory.activated | 0.1591 | 0.033310086 | 1.00E+00 |
| CD3 | cibersort.T.cells.regulatory.Tregs. | -0.0096 | 0.897738439 | 1.00E+00 |
| CD4 | xcell.CD4.memory.T.cells | 0.5856 | 7.31E-18 | 3.80E-16 |
| CD4 | xcell.CD4.naive.T.cells | 0.6153 | 4.92E-20 | 2.56E-18 |
| CD4 | xcell.CD4.T.cells | 0.7052 | 3.10E-28 | 1.61E-26 |
| CD4 | xcell.CD4.Tcm | 0.6062 | 2.42E-19 | 1.26E-17 |
| CD4 | xcell.CD4.Tem | 0.3156 | 1.68E-05 | 8.72E-04 |
| CD4 | cibersort.T.cells.CD4.naive | 0.5275 | 3.27E-14 | 1.70E-12 |
| CD4 | cibersort.T.cells.CD4.memory.resting | 0.1845 | 0.013379321 | 6.96E-01 |
| CD4 | cibersort.T.cells.CD4.memory.activated | 0.1011 | 0.177689557 | 1.00E+00 |
| CD4^+^/CD45RA^+^ | xcell.CD4.memory.T.cells | 0.4838 | 6.80E-12 | 3.54E-10 |
| CD4^+^/CD45RA^+^ | xcell.CD4.Tcm | 0.5603 | 3.44E-16 | 1.79E-14 |
| CD4^+^/CD45RA^+^ | xcell.CD4.Tem | 0.1848 | 0.013252701 | 6.89E-01 |
| CD4^+^/CD45RA^+^ | cibersort.T.cells.CD4.memory.resting | 0.2071 | 0.005387327 | 2.80E-01 |
| CD4^+^/CD45RO^+^ | xcell.CD4.naive.T.cells | 0.4836 | 6.95E-12 | 3.62E-10 |
| CD4^+^/CD45RO^+^ | xcell.CD4.T.cells | 0.6544 | 2.95E-23 | 1.53E-21 |
| CD4^+^/CD45RO^+^ | cibersort.T.cells.CD4.naive | 0.3752 | 2.27E-07 | 1.18E-05 |
| CD8 | xcell.CD8.naive.T.cells | 0.1682 | 0.024394662 | 1.00E+00 |
| CD8 | xcell.CD8.T.cells | 0.6899 | 1.24E-26 | 6.44E-25 |
| CD8 | xcell.CD8.Tcm | 0.6852 | 3.67E-26 | 1.91E-24 |
| CD8 | xcell.CD8.Tem | 0.4848 | 6.05E-12 | 3.15E-10 |
| CD8 | cibersort.T.cells.CD8 | 0.6714 | 8.12E-25 | 4.22E-23 |
| CD8^+^/CD45RA^+^ | xcell.CD8.T.cells | 0.6708 | 9.27E-25 | 4.82E-23 |
| CD8^+^/CD45RA^+^ | xcell.CD8.Tcm | 0.6483 | 1.01E-22 | 5.23E-21 |
| CD8^+^/CD45RA^+^ | xcell.CD8.Tem | 0.4002 | 2.85E-08 | 1.48E-06 |
| CD8^+^/CD45RO^+^ | xcell.CD8.naive.T.cells | 0.0767 | 0.30688712 | 1.00E+00 |
| CD8^+^/CD45RO^+^ | xcell.CD8.T.cells | 0.4621 | 7.39E-11 | 3.84E-09 |
| CD8^+^/CD45RO^+^ | cibersort.T.cells.CD8 | 0.5439 | 3.57E-15 | 1.85E-13 |

**Table S5.** Mean immune cell deconvolution values, p-values, and false discovery rate (FDR) values: cladribine tablets 3.5mg/kg, 5.25 mg/kg, and combined vs placebo. A multivariate linear regression model was built for each cell type to test whether treatment arm is significantly related to the deconvolution score.  Patients’ age and gender were used as covariates in each model. Celltypes for which the adjusted p-values <0.1 for the treatment coefficient ( FDR.Low.vs.Placebo , FDR.High.vs.Placebo, FDR.Treated.vs.Placebo) and adjusted p-values from overall f-test <0.1 were considered   significant. Cladribine tablets treatment FDR values <0.1 for cell signature reductions: blue – downregulated; orange - upregulated.

| **Cell type signatures** | **Placebo_mean** | **LowDose_mean** | **HighDose_mean** | **FDR.FSTAT.Low.vs.Placebo** | **FDR.FSTAT.High.vs.Placebo** | **FDR.FSTAT.Treated.vs.Placebo** | **FDR.Low.vs.Placebo** | **FDR.High.vs.Placebo** | **FDR.Treated.vs.Placebo** |
| --- | --- | --- | --- | --- | --- | --- | --- | --- | --- |
| xcell.aDC | 0.01 | 0.007 | 0.008 | 0.349119217 | 0.14081416 | 0.157315446 | 0.292238471 | 0.423295006 | 0.246126337 |
| xcell.B-cells | 0.13 | 0.117 | 0.14 | 0.471011525 | 0.14081416 | 0.54170341 | 0.372921065 | 0.749892137 | 0.858250838 |
| xcell.Basophils | 0.001 | 0.007 | 0.01 | 0.300551661 | 0.14033777 | 0.119762148 | 0.118161968 | 0.063923862 | 0.069016607 |
| xcell.CD4+ memory T-cells | 0.357 | 0.258 | 0.245 | 3.55E-07 | 2.78E-08 | 1.95E-10 | 2.14E-07 | 1.50E-08 | 3.93E-10 |
| xcell.CD4+ naive T-cells | 0.06 | 0.014 | 0.014 | 5.59E-12 | 3.31E-12 | 1.04E-19 | 3.28E-12 | 2.60E-12 | 1.38E-19 |
| xcell.CD4+ T-cells | 0.229 | 0.141 | 0.131 | 2.51E-10 | 3.31E-12 | 8.76E-16 | 2.14E-10 | 2.60E-12 | 6.89E-15 |
| xcell.CD4+ Tcm | 0.077 | 0.031 | 0.021 | 2.99E-06 | 1.25E-10 | 3.34E-13 | 1.89E-07 | 5.65E-12 | 1.96E-14 |
| xcell.CD4+ Tem | 0.028 | 0.018 | 0.015 | 0.05232528 | 0.02488977 | 0.005045594 | 0.023551204 | 0.006113459 | 0.002133522 |
| xcell.CD8+ naive T-cells | 0.046 | 0.028 | 0.032 | 0.016100773 | 0.14081416 | 0.021346736 | 0.003075785 | 0.043682679 | 0.003607137 |
| xcell.CD8+ T-cells | 0.072 | 0.024 | 0.026 | 5.59E-12 | 1.58E-09 | 8.76E-16 | 2.14E-10 | 2.59E-09 | 2.51E-14 |
| xcell.CD8+ Tcm | 0.092 | 0.049 | 0.053 | 7.54E-08 | 3.94E-06 | 4.98E-10 | 2.91E-06 | 7.12E-05 | 2.29E-07 |
| xcell.CD8+ Tem | 0.029 | 0.024 | 0.026 | 0.201599692 | 0.44864383 | 0.603094829 | 0.625520468 | 0.749892137 | 0.693793128 |
| xcell.cDC | 0.08 | 0.071 | 0.081 | 0.18623134 | 0.42511826 | 0.492101261 | 0.215836993 | 0.749892137 | 0.754711749 |
| cibersort.B.cells.memory | 0.024 | 0.009 | 0.011 | 7.56E-08 | 8.70E-07 | 5.72E-11 | 3.16E-09 | 2.19E-07 | 7.89E-12 |
| cibersort.B.cells.naive | 0.003 | 0.013 | 0.013 | 0.127844395 | 0.03891512 | 0.057641869 | 0.025749853 | 0.025627693 | 0.012145391 |
| cibersort.Dendritic.cells.activated | 0 | 0.00E+00 | 0.00E+00 | 0.517304952 | 0.75602288 | 0.714612264 | 0.559673193 | 0.712264466 | 0.548874794 |
| cibersort.Dendritic.cells.resting | 0.003 | 0.001 | 0.001 | 0.000189477 | 7.26E-05 | 6.44E-07 | 0.003101862 | 8.31E-05 | 1.27E-05 |
| cibersort.Eosinophils | 0.004 | 0.002 | 0.001 | 0.192827825 | 0.07228828 | 0.068498497 | 0.247333111 | 0.072823903 | 0.033242695 |
| cibersort.Macrophages.M0 | 0.002 | 0.004 | 0.003 | 0.18623134 | 0.26719544 | 0.073299784 | 0.413657014 | 0.530940222 | 0.422938552 |
| cibersort.Macrophages.M1 | 0 | 0 | 0 | 0.057040192 | 0.14081416 | 0.023352671 | 0.099543583 | 0.086051244 | 0.025928823 |
| cibersort.Macrophages.M2 | 0.032 | 0.035 | 0.034 | 0.349829 | 0.37644618 | 0.291691356 | 0.101939276 | 0.255366693 | 0.108181937 |
| cibersort.Mast.cells.resting | 0.066 | 0.063 | 0.069 | 0.638400705 | 0.75602288 | 0.96304359 | 0.547004121 | 0.423295006 | 0.864446752 |
| cibersort.Monocytes | 0.059 | 0.065 | 0.071 | 0.635432224 | 0.00210584 | 0.005398304 | 0.277561806 | 0.009241702 | 0.019441627 |
| cibersort.Neutrophils | 0.37 | 0.429 | 0.411 | 0.000169568 | 0.00771371 | 0.000264726 | 0.001785188 | 0.063923862 | 0.006231679 |
| cibersort.NK.cells.activated | 0.061 | 0.055 | 0.064 | 0.551482712 | 0.9118721 | 0.989266852 | 0.372921065 | 0.705426005 | 0.864446752 |
| cibersort.NK.cells.resting | 0.104 | 0.097 | 0.103 | 0.107657912 | 0.07508635 | 0.023352671 | 0.663336144 | 0.901184448 | 0.93699034 |
| cibersort.Plasma.cells | 0.009 | 0.006 | 0.007 | 0.05034369 | 0.01406191 | 0.006778713 | 0.009528453 | 0.005051934 | 0.001182188 |
| cibersort.T.cells.CD4.memory.activated | 0.018 | 0.016 | 0.016 | 0.300551661 | 0.75602288 | 0.505257779 | 0.412864185 | 0.337357618 | 0.241948829 |
| cibersort.T.cells.CD4.memory.resting | 0.013 | 0.006 | 0.006 | 0.403236923 | 0.15894215 | 0.120696091 | 0.142236288 | 0.107539898 | 0.037082005 |
| cibersort.T.cells.CD4.naive | 0.089 | 0.065 | 0.06 | 7.49E-05 | 3.07E-10 | 7.16E-11 | 4.32E-06 | 3.48E-10 | 2.52E-11 |
| cibersort.T.cells.CD8 | 0.145 | 0.131 | 0.13 | 0.110490353 | 0.02549261 | 0.026076115 | 0.414732738 | 0.525715631 | 0.419703281 |
| cibersort.T.cells.regulatory..Tregs. | 0 | 0 | 0 | 0.380998755 | 0.34413263 | 0.124615858 | 0.511181599 | 0.344510067 | 0.419703281 |
| xcell.Class-switched memory B-cells | 0.086 | 0.065 | 0.073 | 0.053767296 | 0.02181489 | 0.025409414 | 0.009084798 | 0.024118038 | 0.005576836 |
| xcell.CLP | 0.13 | 0.1 | 0.108 | 0.000189477 | 0.01786859 | 0.000264726 | 0.000903386 | 0.036218102 | 0.002031605 |
| xcell.CMP | 0.013 | 0.02 | 0.02 | 0.127538522 | 0.00946184 | 0.004186869 | 0.099543583 | 0.071416731 | 0.040238812 |
| xcell.DC | 0.016 | 0.018 | 0.02 | 0.663593732 | 0.36590151 | 0.664297723 | 0.663336144 | 0.238244689 | 0.358166216 |
| xcell.Eosinophils | 0.29 | 0.36 | 0.329 | 0.016811429 | 0.17242759 | 0.062457412 | 0.009084798 | 0.117835299 | 0.018234616 |
| xcell.Erythrocytes | 0.137 | 0.175 | 0.178 | 0.016959523 | 0.01406191 | 0.004655814 | 0.02347876 | 0.007811435 | 0.002257759 |
| xcell.GMP | 0.043 | 0.032 | 0.034 | 0.053767296 | 0.06555699 | 0.019894775 | 0.23842481 | 0.437208112 | 0.234193127 |
| xcell.HSC | 0.024 | 0.026 | 0.022 | 0.945392601 | 0.82188666 | 0.706973187 | 0.625520468 | 0.785760963 | 0.951880134 |
| xcell.iDC | 0.025 | 0.035 | 0.028 | 0.212149531 | 0.8458261 | 0.355263026 | 0.048892993 | 0.543959669 | 0.169759807 |
| xcell.Macrophages | 0.025 | 0.036 | 0.04 | 0.180255987 | 0.01406191 | 0.004186869 | 0.103486482 | 0.009126817 | 0.007590068 |
| xcell.Macrophages M1 | 0.006 | 0.008 | 0.006 | 0.839956995 | 0.79619062 | 0.989266852 | 0.663336144 | 0.979119602 | 0.850684088 |
| xcell.Macrophages M2 | 0.024 | 0.036 | 0.041 | 0.110490353 | 0.00309538 | 0.000523497 | 0.068821971 | 0.006113459 | 0.004166968 |
| xcell.Mast cells | 0.009 | 0.013 | 0.014 | 0.201843126 | 0.0088275 | 0.00390578 | 0.099543583 | 0.007811435 | 0.007590068 |
| xcell.Megakaryocytes | 0.166 | 0.215 | 0.205 | 0.00072789 | 0.02130061 | 0.000264726 | 0.010487328 | 0.086051244 | 0.012793435 |
| xcell.Memory B-cells | 0.04 | 0.045 | 0.056 | 0.37922348 | 0.10452618 | 0.305991064 | 0.824459434 | 0.159183083 | 0.329566421 |
| xcell.MEP | 0.009 | 0.002 | 0.004 | 0.001609999 | 0.09195244 | 0.004549636 | 0.006200836 | 0.094828628 | 0.006231679 |
| xcell.Monocytes | 0.00E+00 | 0.00E+00 | 0 | 0.663593732 | 0.72221249 | 0.749428556 | 0.971716663 | 0.522467824 | 0.667805792 |
| xcell.MPP | 0.061 | 0.075 | 0.073 | 0.09260778 | 0.07508635 | 0.006929296 | 0.103486482 | 0.203414535 | 0.074477082 |
| xcell.MSC | 0.108 | 0.118 | 0.122 | 0.523460055 | 0.68296891 | 0.496873592 | 0.666310949 | 0.531819371 | 0.549828438 |
| xcell.naive B-cells | 0.072 | 0.078 | 0.091 | 0.606118041 | 0.06109027 | 0.291691356 | 0.704473329 | 0.147269099 | 0.303248389 |
| xcell.Neutrophils | 0.027 | 0.047 | 0.049 | 0.000820673 | 0.00191007 | 0.000264726 | 0.007202541 | 0.001395992 | 0.00057634 |
| xcell.NK cells | 0.022 | 0.017 | 0.023 | 0.090296388 | 0.8458261 | 0.427218573 | 0.666310949 | 0.749892137 | 0.987994832 |
| xcell.NKT | 0.164 | 0.237 | 0.228 | 0.000231797 | 0.00023349 | 7.83E-06 | 0.000526252 | 0.002513694 | 8.41E-05 |
| xcell.pDC | 0.002 | 0.001 | 0.001 | 0.349829 | 0.17970476 | 0.142885004 | 0.427505723 | 0.836315554 | 0.549828438 |
| xcell.Plasma cells | 0.038 | 0.019 | 0.022 | 0.000151217 | 0.00024038 | 7.83E-06 | 0.000145731 | 0.00178163 | 1.77E-05 |
| xcell.Platelets | 0.103 | 0.115 | 0.116 | 0.053767296 | 0.17995224 | 0.02467415 | 0.18764729 | 0.218761157 | 0.12284898 |
| xcell.pro B-cells | 0.001 | 0.001 | 0.003 | 0.635432224 | 0.42694185 | 0.622131308 | 0.625520468 | 0.122489834 | 0.234193127 |
| xcell.Tgd cells | 0.053 | 0.038 | 0.039 | 0.001000125 | 0.00218132 | 0.000264726 | 0.007202541 | 0.024006614 | 0.005475385 |
| xcell.Th1 cells | 0.09 | 0.089 | 0.093 | 0.955465422 | 0.43361052 | 0.370228323 | 0.859002181 | 0.959355747 | 0.999162934 |
| xcell.Th2 cells | 0.047 | 0.035 | 0.034 | 0.038614133 | 0.00191007 | 0.000264726 | 0.039136693 | 0.054223488 | 0.013328445 |
| xcell.Tregs | 0.118 | 0.106 | 0.092 | 0.349119217 | 0.01406191 | 0.026076115 | 0.22896967 | 0.004836539 | 0.011423278 |

**Figure S1. Scatter plots of cell counts and CIBERSORT signatures for GSE93777 set. All data points were taken into account.**


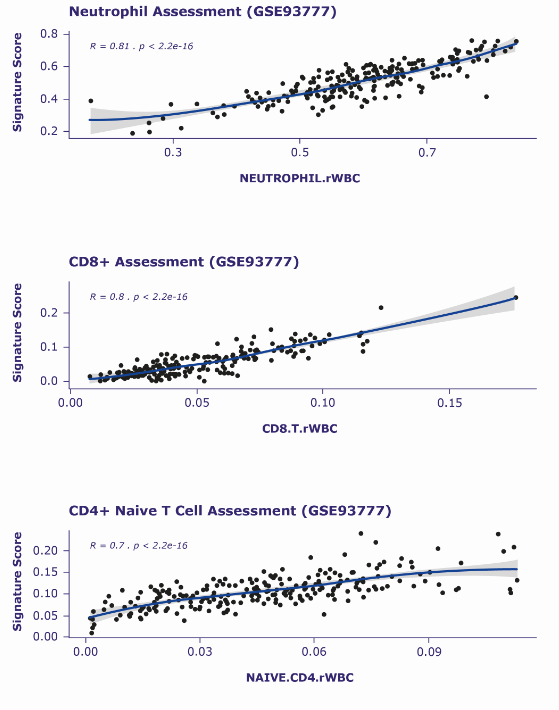


**Figure S2. Online deconvolution and flow cytometry tool:** [**https://emdserono1.shinyapps.io/Immune_Cell_Deconvolution_Validation/**](https://emdserono1.shinyapps.io/Immune_Cell_Deconvolution_Validation/)


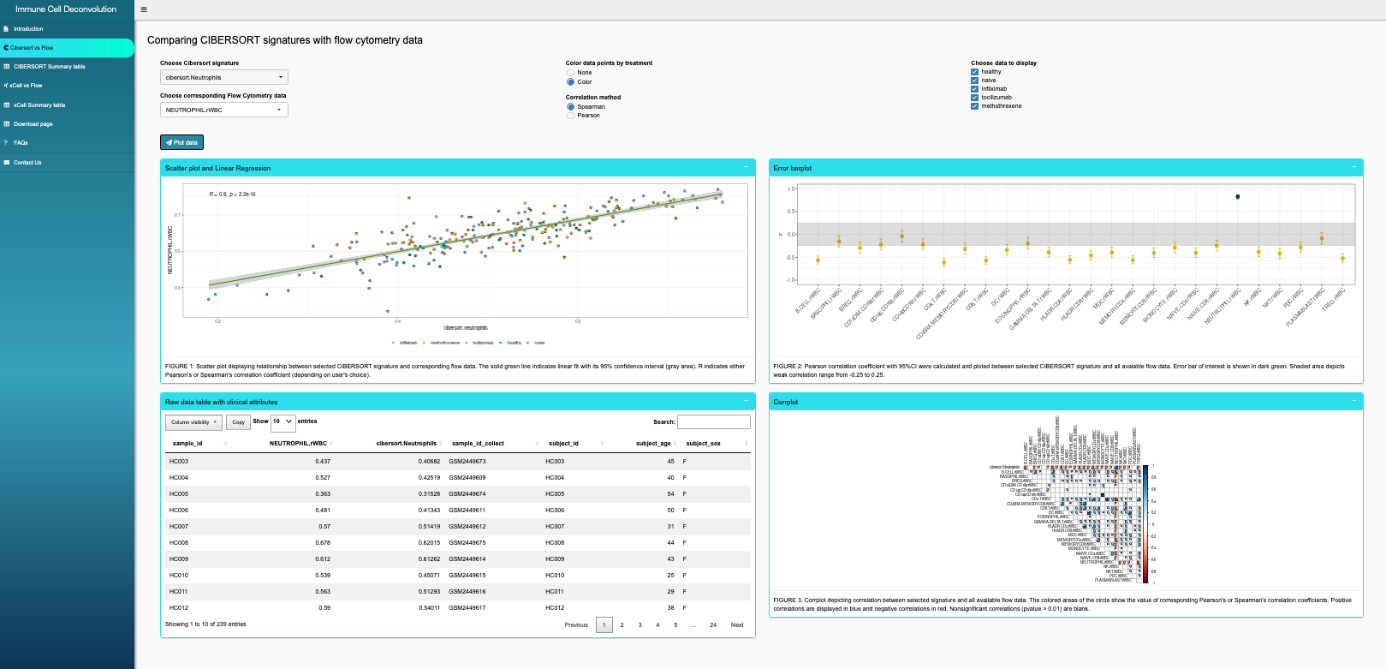

Supplement: Supplementary file 1 — Supplementary Information 1. [file 41598_2023_34384_MOESM1_ESM.docx]
