# Supplementary material for: Revealing the immune cell subtype reconstitution profile in patients from the CLARITY study using deconvolution algorithms after cladribine tablets treatment
Source: Sci Rep. 2023 May 18;13:8067. doi: 10.1038/s41598-023-34384-5 (PMC10193326; doi:10.1038/s41598-023-34384-5)
Supplement: Supplementary file 3 — Supplementary Information 3. [file 41598_2023_34384_MOESM3_ESM.pdf]

Site #	PI	IRB/EC	IRB/EC Chairperson	Country
002	John King	Melbourne Health Human Research Ethics Committee		Australia
003	John Pollard	SSWAHS Ethics Review Committee (RPAH Zone)		
004	Leslie Sedal	St Vincent's Hospital (Melbourne) STV HREC		
All	All	Central EC: EC of the Medical University of Vienna	Ernst Singer	Austria
005	Franz Aichner	Local EC: EC of the country Upper Austria Landesnervenklinik Wagner-Jauregg	Johannes Fischer	
006	Christian Eggers	Local EC: EC of the Hospital Barmherzige Brüder Linz	Kurt Lenz	
009	Robert Medaer	Local EC: Comité d'Ethique Hospitalo-Facultaire - CHU Sart Tilman	Maurice Lamy	Belgium
008	Dominique Dive	Leading EC: Commissie voor Medische Ethiek, Universiteit Hasselt,	Creemers	
10	Maria Lucia Brito Ferreira	CEP/HR Av. Agamenon Magalhães, s/n - 5 andar (floor), Derby - Recife - PE - Brazil - 52020-000	Josimario Silva	Brazil
014	Nadezhda Deleva	UMBAL "St.Marina"	Marinka Peneva	Bulgaria
204	Ivaylo Petrov	MBAL Shumen AD	Magdalena Dimitrova Angelova	
015	Lyubomir Haralanov	SBAL SSZ NKB	V. Petrov	
205	Neli Petrova	MBAL - Ruse	Grigory Lefterov	
019	Boyko Stamenov	UMBAL Pleven	Iavor Ivanov	
016	Ivan Milanov	SBAL NP St.Naum	Pencho Kolev	

Site #	PI	IRB/EC	IRB/EC Chairperson	Country
017	Radostin Moskov	Local EC: Military Medical Academy		Bulgaria
018	Penko Shotekov	UMHAT "Alexandrovska"		
020	Zahari Zahariev	University Multiprofile for Active Treatment "Sr Georgi" -Plovdiv	Blagovest Pehlivanov	
203	Ivan Manchev	UMBAL - Stara Zagora EAD	Yovcho Yovchev	
013	Stoyan Bozhinov	university Multiprofile Hospital for Active Treatment - Dr George Stranski -Pleven EAD	Iavor Ivanov	
21	Mark Freedman	The Ottawa Hospital, 725 Parkdale Avenue, Civic Campus, Ottawa, K1Y 4E9, Ontario	Francine Sarazin	Canada
22	Francois Grand'Maison	Comite dethique de la recherche Hopital Charles LeMoyne 3120 boul Taschereau Greenfield Park, Quebec J4V 2 H1 Canada	Sylvain Brunet	
23	Francois Jacques	Comite d"ethique de la recherche, 909 Boul. de la Verendrye Ouest, Gatineau, J8Y 1W7, Quebec	Me Jacques Maziade	
28	Galina Vorobeychik			
141	François Émond	Comite d'Ethique de la recherche de l'Hopital Enfant-Jesus du CHA, 1401 - 18eme rue, Quebec, G1J 1Z4, Quebec	Me Jacques Maziade	
For all sites		Central EC: Multicentric Ethics Committee on Clinical Trial on Human Medicinal Products of the University Hospital Motol	Vratislav Smelhaus	Czech Republic
030	Eva Havrdova	local EC: Lokální EK VFN Praha	Josef Šedivý	
181	Petr Kanovsky	local EC:Etická komise FN Olomouc a LF v Olomouci	Vladko Horčička	
031	Radomir Talab	local EC:Etická komise FN Hradec Králové	Jiří Vortel	

Site #	PI	IRB/EC	IRB/EC Chairperson	Country
197	Vida Demarin	Central EC: Agency of Medicinal products and Medical devices	Prof. Dinko Vitezic	Croatia
198	Tatjana Perhat-Bucevic			
199	Jasna Bozic			
201	Ivo Lusic			
033	Soelberg Sørensen	The Scientific Etics Commitees-Capital region	Birgit Tystrup	Denmark
032	Thor Petersen			
035	Irja Kalbe	Ethics Review Committee (ERC) on Human Research of the University of Tartu	Andres Soosaar	Estonia
034	Katrin Gross-Paju			
036	Toomas Toomsoo			
037	Irina Elovaara	Ethics Committee of Hospital District of Southwest Finland	Mika Scheinin	Finland
038	Juha-Pekka Eralinna			
041	Mauri Reunanen			
047	Gilles Edan	Comité de Protection des Personnes Nord Ouest IV<br>Service de Pharmacologie<br>Faculté de Médecine - Pôle Recherche	P.Y Hatron	France
046	Marc Debouverie			
050	Patrick Vermersch			
044	Pierre Clavelou			
138	Pierre Labauge	Comité de Protection des Personnes Nord Ouest IV<br>Service de Pharmacologie<br>Faculté de Médecine - Pôle Recherche	Jouet	

Site #	PI	IRB/EC	IRB/EC Chairperson	Country
136	Philippe Damier	Comité de Protection des Personnes Nord Ouest IV<br>Service de Pharmacologie<br>Faculté de Médecine - Pôle Recherche		France
137	Olivier Gout	Comité de Protection des Personnes Nord Ouest IV<br>Service de Pharmacologie<br>Faculté de Médecine - Pôle Recherche		
064	Maria Pia Amato	Comitato Etico Per La Sperimentazione Farmacologica Dell'Azienda Ospedaliera Careggi<br>Viale Pieraccini 28<br>50139 Firenze		Italy
065		Comitato Etico Dell'Azienda Ospedaliera Universitaria Poloclinico Tor Vergata<br>Viale Oxford 81<br>00133 Roma		
066		Comitato Etico Per Le Attivita´ Biomediche Dell´Univerita´ Degli Studi Federico II Di Napoli<br>Indirizzo: via Pansini 5<br>80131 Napoli		

Site #	PI	IRB/EC	IRB/EC Chairperson	Country
067	Giancarlo Comi	Comitato Etico Della Fondazione Centro S. Raffaele Del Monte Tabor (IRCCS) Di Milano I<br>Via Olgettina 60<br>20132 Milano		Italy
068		Comitato Etico Per La Sperimentazione Dell'Azienda Ospedaliera Di Padova - Veneto<br>Via Giustiniani 2<br>35128 Padova		
069		Comitato Etico Dell`Azienda Ospedaliera San Camillo - C. Forlanini Di Roma<br>Via Portuense, 332<br>00149 Città: Roma		
070	Maria Marrosu	Comitato Etico<br>Azienda USL 8 Cagliari<br>Via Piero della Francesca, 1<br>09047 Selargius Cagliari<br>Numero telefonico: 070 609 3632		
071		Comitato Bioetico Dell'Azienda Policlinico Dell'Universita' Di Catania - Sicilia<br>Via S.Sofia, 78<br>95123 Catania		
072		Comitato Etico<br>Azienda Ospedaliera Sant'Andrea<br>Via di Grottarossa 1035 - 00185 Roma		

Site #	PI	IRB/EC	IRB/EC Chairperson	Country
073	Maria Trojano	Comitato Etico Dell`Azienda Ospedaliera (IEC) - Ospedale Policlinico Consorziale Di Bari -Bari<br>Piazza G. Cesare, 11<br>70124 Bari		Italy
161		Comitato Etico<br>Azienda Ospedaliera Universitaria S. Martino<br>L.go Rossana Benzi, 10<br>16132 Genova		
056		Ethik-Kommission der Medizinischen Fakultät der Universität Regensburg	PD Dr. Jörg Marienhagen	Germany
054	Mathias Maurer / Heinz Wiendl / Christoph Kleinschnitz	Ethik-Kommission der Medizinischen Fakultät der Universität Würzburg	Prof. Dr. med. Dr. phil. M Stolberg	
055	Sebastian Klaus Schimrigk / Andrew Chan	Ethik-Kommission der Medizinischen Fakultät der Ruhr-Universität Bochum	Prof. Dr. M. Zenz	
053	Volker Limmroth / H.-C. Diener	Ethik-Kommission an der Medizinischen Fakultät der Universität Duisburg-Essen	Prof. Dr. Karl H. Jakobs	
139	Patrick Oschmann / Kerstin Retzlaff	Ethik-Kommission des Fachbereiches Humanmedizin Universitätsklinikum Gießen und Marburg GmbH	Prof. Dr. K. L. Schmidt	
052	Bernd Kieseier	Ethik-Kommission der Medizinischen Fakultät der Heinrich-Heine-Universität Düsseldorf	Prof. Dr. med. Hans-Gerd Lenard	
051	Fedor Heidenreich	Ethik-Kommission der Medizinischen Hochschule Hannover	Prof. Dr. H.D. Troger	
133	Ulf Ziemann	Ethik-Kommission des Fachbereichs Medizin der Johann-Wolfgang-Goethe-Universität Frankfurt	Prof. Dr. med. Sebastian Harder	

Site #	PI	IRB/EC	IRB/EC Chairperson	Country
057	Uwe Zettl	Ethik-Kommission an der Medizinische Fakultät der Universität Rostock	Prof. Dr. med Rudolf Wegener	Germany
060	Alexander Papadimitriou	Scientific Council of Henry Dynant Hospital	Ioannis Papadimitriou	
058	Athanassios Kyritsis	Scientific Council of University General Hospital of Ioannina	Ioannis Goudevenos	
202	Klimentini Karageorgiou	Scientific Council of General Hospital of Athens “G. Gennimatas”	Antonios Asimakopoulos	
174	Maija Metra	Ethics Committee for Clinical Trial<br>Zaku kliniskas izpetes Etikas komiteja	Nilss Poritis	Latvia
074	Salam Koussa	Comite d'Ethique de l'Hotel Dieu de France, Beyrouth - Liban	Georges Halaby	Lebanon
075	Bassem Yamout	IRB - Faculty of Medicine - American University of Beirut - Lebanon	Ibrahim Salti	
151	Souheil Gebeily	Comite d'Ethique de l'Hopital Libanais Geitawi, Beyrouth - Liban	Jose Khabouth	
175	Mohamed Wehbi	Ethics Committee of Sahel General Hospital - Ghobeiry, Beirut - Lebanon	Ibrahim Deghaim	
182	Antanas Vaiikus	Lithuanian Bioethics Committee	Eugenijus Gefenas	Lithuania
176	Mohamed Yahyaoui	Comité d'éthique de la Recherche Biomédicale, Université Mohammed V, Souissi, Faculté de Médecine et de Pharmacie de Rabat	Jamal Eddine Ktiouet	Morocco
177	Rachid Mosseddaq			
178	Ilham Slassi	Faculté de médecine et de pharmacie de Casablanca<br>Centre hospitalier universitaire Bnou Rochd<br>Comité d'éthique pour la Recherche biomédicale	Farid Hakkou	
179	Ouafae Messouak	Comité d'éthique de la Recherche Biomédicale, Université Mohammed V, Souissi, Faculté de Médecine et de Pharmacie de Rabat	Jamal Eddine Ktiouet	

Site #	PI	IRB/EC	IRB/EC Chairperson	Country
077	Peter Joseph Hubert Jongen	Academisch Ziekenhuis Maastricht, Medisch Ethische Toetsingscommissie		Netherlands
076	Raymond Hupperts			
078	Anna Czlonkowska	Komisja Bioetyki Uniwersytetu Medycznego w Łodzi	Przedzisław Polakowski	Poland
082	Andrzej Szczudlik			
081	Krzysztof Selmaj			
080	Walenty Nyka		Przedzisław Polakowski & Professor Kennedy Lees	
079	Wojciech Kozubski		Przedzisław Polakowski	
083	José Figueiredo	Comissão de Ética para a Investigação Clínica	António Barros Veloso	Portugal
084	Rui Pedrosa			
all sites	all Russian investigators	The Ethics Committee under the Federal Service on Surveillance in Healthcare and Social Development	Fedor Komarov	Russian Federation
all sites	all Russian investigators	The Independent Interdisciplinary Committee for Ethical Expertise of Clinical Studies	Konstantin Tebloev	
085	Valentina Alifirova	Ethics Committee of the Siberian State Medical University	Elena Idrisova	
086	Victor Balyazin	The Ethics Committee of the Rostov State Medical University	Vladimir Terentyev	
088	Anna Belova	Independent Committee of Ethic of Municipal Medical Unit "City Hospital #33"	Mikhail Rizhov	
089	Alexei Boiko	Ethics Committee of Moscow State Institution of Health Care	Eugeniy Shmelyov	

Site #	PI	IRB/EC	IRB/EC Chairperson	Country
090	Alexander Elchaninov	Ethics Committee of 122 Central Medical Clinic of the Ministry of Health of Russian Federation	Dmitry Tikhomirov	Russian Federation
092	Edouard Jakoupov	Republican Committee on Ethic questions in conducting clinical trials-studies of medical products under Ministry of Health of Republic of Tatarstan	Alexey Sozinov	
093	Sergey Kotov	Local Ethic Committee at Vladimirsky Clinical Institute	Vasily Isakov	
094	Olga Lesnyak	Ethics Committee of the State institution of Public Health “Sverdlovsk Regional Clinical Hospital # 1”	Eugeniya Rozhdestvenskaya	
096	Miroslav Odinak	The Ethics Committee of the Kirov Military Medical Academy	Yury Lobzin	
098	Irina Poverennova	Bioethical committee at Samara State Medical University	Larisa Volova	
099	Alexander Skoromets	Ethics Committee of the Pavlov State Medical University	Nikolay Neznanov	
100	Nikolay Spirin	Ethic Committee of Yaroslavl State Medical Academy	Alexey Pavlov	
101	Igor Stolyarov	The Ethics Committee of the Institute of the Human Brain	Sergey Danko	
102	Olga Vorobeva	Ethic Committee at Central Clinical Military Hospital of Federal Security Service of Russian Federation	Natalia Obidina	
103	Olga Voskresenskaya	Ethic Committee of Saratov State Medical University	Elena Kashkina	

Site #	PI	IRB/EC	IRB/EC Chairperson	Country
104	Leonid Zaslavaskiy	The Local Ethics Committee of the Leningrad Regional Hospital	Galina Rivkina	Russian Federation
105	Elena Zonova	Ethics Committee of the Institute Clinical and Experimental Lymphology SB RAMS	Elena Letyagina	
171	Anatoliy Kudryavtsev	Local Ethics Committee at Vladimir State Healthcare Institution "Vladimir Regional Clinical Hospital"	Galina Barabashkina	
173	Nikolay Zhulev	The Ethics Committee of the Saint-Petersburg Medical Academy of Postgraduate Education	Inna Gavrisheva	
186	Saeed Bohlega	King Faisal Specialist Hospital & Research Centre	Sultan Al Sedairy	Saudi Arabia
187	Mohammed Al Jumah	Institutional Review Board Committee, National Guard Health Affairs, King Abdulaziz Medical City	Soud Al Rasheed	
106	Jelena Drulovic	Ethics Committee of Clinical Center of Serbia	Goran Milosinovic	Serbia
107	Congor Nadj	Ethics Committee of Clinical Center of Vojvodina	Slobodan Curic	
114	Norbert Goebels	Ethikkommission des Kantons Zürich (KEK)	Niklaus Herzog	Switzerland
115	Myriam Schluep	Commission d'Ethique de la Recherche Clinique Lausanne	Jean-Patrice Gardaz	
144	Barbara Tettenborn	Ethikkommission des Kantons St. Gallen (EKSG)	Georg Kreienbühl	
166	Chokri Mhiri	Ethic commitee of Hôpital Bourguiba		Tunisia
167	Faycal Hentati	Ethic commitee of Institut National de Neurologie La Robta		
168	Ridha Mrissa	Ethic commitee of Hôpital Militaire de Tunis		
184	Mahbouba Ayed	Ethic commitee of Hôpital Fattouma Bourghiba		
185	Amel Mrabet	Ethic commitee of Hôpital Charles Nicolle		

Site #	PI	IRB/EC	IRB/EC Chairperson	Country
117	Egemen Idiman	Hacettepe Üniversitesi Tıp Fakültesi Tıbbi Araştırmalar Yerel Etik Kurulu--Hacettepe University Faculty Of Medicine Medical Research Local Ethics Committee	Rüştü Onur	Turkey
119	Rana Karabudak	Dokuz Eylül Üniversitesi Tıp Fakültesi İlaç Araştırmaları Yerel Etik Kurulu--Dokuz Eylül University Faculty of Medicine Drug Research Local Ethics Committee	Yeşim TUNÇOK	
121	Omer Faruk Turan	Uludağ Üniversitesi Tıp Fakültesi Tıbbi Araştırmalar Etik Kurulu-- Research Ethics Committee of Medical Faculty,Uludag University	Levent Buyuksakal	
127	Konstantin Loganovsky	Cetral Ethics Commission of the Ministry of Health of Ukraine	Vasyl Kornatskyy	Ukraine
128	Tetyana Nehrych			
130	Nataliya Voloshyna			
129	Sergiy Moskovko			
126	Basil Sharrack	Multicentre Research Ethics Committee for Scotland A / MREC for Scotland	Lees Kennedy	United Kingdom
124	Clive Hawkins			
122	Cris Constantinescu			
132	Fayyaz Ahmed			
123	Gavin Giovannoni			
125	Jacqueline Palace			

Site #	PI	IRB/EC	IRB/EC Chairperson	Country
143	Stuart Cook	Western Institutional Review Board 3535 Seventh Avenue, SW I Olympia, WA 98508-2029	Theodore Schultz	US
145	Barrie Hurwitz	Duke University Health System Institutional Review Boar Hock Plaza 2424 Erwin Road, Suite 405 Campus Box 2712 Durham, NC 27705	John Harrelson, MD	
146	Walter Royal	University of Maryland School of Medicine Human Research Protections Office UMB Biopark 1 800 West Baltimore Street, Suite 100 Baltimore, MD 21201	Robert Edelman, MD	
147	Kiren Kresa-Reahl	Coast Independent Review Board 5475 Mark Dabling Blvd Suite 351 Colorado Springs, CO 80918	Melissa Cortes	
148	Gabriel Pardo	Mercy Health System's Review Board, 4300 W. Memorial Road Oklahoma City, Oklahoma 73120	Linda Fanning	
150	Daniel Wynn	Coast Independent Review Board 5475 Mark Dabling Blvd Suite 351 Colorado Springs, CO 80918	Melissa Cortes	
153	Walter Carlini			
154	George Garmany Jr.			
156	Daniel Mikol	IRB MED Argus 1 517 W. William Ann Arbor, Michigan 48103-4943	John Weg, MD	
157	T. Hemanth Rao	Coast Independent Review Board 5475 Mark Dabling Blvd Suite 351 Colorado Springs, CO 80918	Melissa Cortes	
158	Dusan Stefoski	IRB at Rush-Prebyterian St. Luke's Medical Center 1725 W. Harrison, Suite 439 Chicago, IL 60612	Allen Korenblit M.D., C.I.P	

Site #	PI	IRB/EC	IRB/EC Chairperson	Country
159	Mary Reif	Coast Independent Review Board 5475 Mark Dabling Blvd Suite 351 Colorado Springs, CO 80918	Melissa Cortes	US
160	Ben Thrower	Shepherd Center, Inc 2020 Peachtree Road, NW Atlanta, George 30309	Michael L. Jones	
163	Kottil Rammohan	Western Institutional Review Board 3535 Seventh Avenue, SW I Olympia, WA 98508-2029	Theodore Schultz	
165	Steven Glyman			
170	Jeffrey English	Coast Independent Review Board 5475 Mark Dabling Blvd Suite 351 Colorado Springs, CO 80918	Melissa Cortes	
207	John Huddlestone			
209	Randall Webb			
